# Contact tracing versus facility-based screening for active TB case finding in rural South Africa: A pragmatic cluster-randomized trial (Kharitode TB)

**DOI:** 10.1371/journal.pmed.1002796

**Published:** 2019-04-30

**Authors:** Colleen F. Hanrahan, Bareng A. S. Nonyane, Lesego Mmolawa, Nora S. West, Tsundzukani Siwelana, Limakatso Lebina, Neil Martinson, David W. Dowdy

**Affiliations:** 1 Department of Epidemiology, Johns Hopkins Bloomberg School of Public Health, Baltimore, Maryland, United States of America; 2 Department of International Health, Johns Hopkins Bloomberg School of Public Health, Baltimore, Maryland, United States of America; 3 Perinatal HIV Research Unit, Soweto, South Africa; University of Cape Town, SOUTH AFRICA

## Abstract

**Background:**

There is a dearth of comparative effectiveness research examining the implementation of different strategies for active tuberculosis (TB) case finding, particularly in rural settings, which represent 60% of the population of sub-Saharan Africa.

**Methods and findings:**

We conducted a pragmatic, cluster-randomized comparative effectiveness trial of two TB case finding strategies (facility-based screening and contact tracing) in 56 public primary care clinics in two largely rural districts of Limpopo Province, South Africa. In the facility-based screening arm, sputum Xpert MTB/RIF was performed on all patients presenting (for any reason) with TB symptoms to 28 study clinics, and no contact tracing was performed. In the contact-tracing arm, contacts of patients with active TB were identified (via household tracing in 14 clinics and using small monetary incentives in the other 14 clinics), screened for TB symptoms, and offered Xpert MTB/RIF testing. The primary outcome was the number of newly identified patients with TB started on treatment. The analysis used multivariable Poisson regression adjusted for historical clinic-level TB case volumes and district. The trial was registered with ClinicalTrials.gov (NCT02808507). From July 18, 2017, to January 17, 2019, a total of 3,755 individuals started TB treatment across 56 study clinics in the 18-month period. Clinic characteristics and clinic-level averages of patient characteristics were similar across the two arms: 40/56 (71%) clinics were in a rural location, 2,136/3,655 (58%) patients were male, and 2,243 (61%) were HIV positive. The treatment initiation ratio comparing the yield of TB patients started on treatment in the facility-based arm compared to that from the contact-tracing arm was 1.04 (95% confidence interval [CI] 0.83–1.30, *p* = 0. 73). In the contact-tracing arm, 1,677 contacts of 788 new TB index patients were screened, yielding 12 new patients with TB. Prespecified subgroup analyses resulted in similar results, with estimated treatment initiation ratios of 0.96 (95% CI 0.64–1.27; *p* = 0.78) and 1.23 (95% CI 0.87–1.59; *p* = 0.29) among historically smaller and historically larger clinics, respectively. This ratio was 1.02 (95% CI 0.66–1.37; *p* = 0.93) and 1.08 (95% CI 0.74–1.42; *p* = 0.68) in the Vhembe and Waterberg districts, respectively. The estimated treatment initiation ratio was unchanged in sensitivity analyses excluding 24 records whose TB registration numbers could not be verified (1.03, 95% CI 0.82–1.29; *p* = 0.78) and excluding transfers-in (1.02, 95% CI 0.80–1.29; *p* = 0.71). Study limitations include the possibility of imbalance on cluster size owing to changes in catchment population over time and the inability to distinguish the independent effects of the two contact investigation strategies.

**Conclusions:**

Contact tracing based on symptom screening and Xpert MTB/RIF testing did not increase the rate of treatment initiation for TB relative to the less resource-intensive approach of facility-based screening in this rural sub-Saharan setting.

**Trial registration:**

ClinicalTrials.gov NCT02808507.

## Abstract 2

In this pragmatic cluster randomized controlled trial, Colleen Hanrahan and colleagues examine the effectiveness of two active case finding strategies for tuberculosis

## Author summary

**Why was this study done?:**

- Active case finding for tuberculosis (TB) is recommended by the World Health Organization as an approach to find patients with previously unidentified TB, particularly in high-burden settings.
- Few studies have compared the yield of feasible strategies to implement active TB case finding in rural settings.

**What did the researchers do and find?:**

- We conducted a cluster-randomized controlled trial examining the effectiveness of two active case finding strategies for TB in two largely rural districts of South Africa: facility-based case finding (screening all attendees at public primary healthcare clinics for TB symptoms and testing patients with symptoms using Xpert MTB/RIF) and contact tracing (testing symptomatic contacts of newly diagnosed TB patients using Xpert MTB/RIF).
- A total of 1,929 TB patients started TB treatment in 28 clinics randomized to facility-based screening, and 1,726 started treatment in 28 size-matched clinics randomized to contact tracing; this corresponds to a treatment initiation ratio of 1.06 (95% confidence interval [CI] 0.83–1.30, *p* = 0.70) comparing facility-based screening to contact tracing.
- In the contact-tracing arm, 1,677 contacts of 789 new TB index patients were screened for TB, yielding 12 new TB diagnoses.

**What do these findings mean?:**

- Feasibly delivered contact tracing, using Xpert MTB/RIF as the diagnostic test, did not increase the clinic-level TB treatment initiation rate above the current standard of care (facility-based screening).
- This finding is driven by lower-than-expected yield from contact tracing, suggesting that although close contacts of patients newly diagnosed with TB patients do have higher-than-average TB risk, higher-sensitivity testing approaches may be necessary to identify TB among this group and increase TB treatment initiation rates at the clinic level.
- Potential limitations of this study include the possibility of changing clinic populations over time, the use of two different contact investigation strategies, and limited generalizability outside of rural sub-Saharan Africa.

## Introduction

Tuberculosis (TB) is the world’s leading infectious disease killer, causing 10 million new cases and 1.3 million deaths in 2017 [1]. TB case detection has languished at near two-thirds for over a decade—in 2017, an estimated 3.6 million cases went unreported or undetected [1]. Evidence from large-scale studies of intensive active case finding strategies in high-burden settings have shown that approaches such as community-wide case finding campaigns, household visits, and contact tracing identify more new patients with TB compared to traditional passive case finding and have a population-level impact, lowering TB prevalence [2, 3]. Investigation of household and other close contacts has been a pillar of TB control activities in high-income, low-prevalence settings for decades [4, 5] but, despite global guidelines in place since 2012 [6], has been poorly implemented in low- and middle-income, high-burden settings. In order to meet ambitious global targets of reducing TB incidence and mortality by 90% by 2035, WHO recommends that ≥90% of contacts of new patients with TB should be investigated for TB [1].

In sub-Saharan Africa, 60% of the population still lives in rural areas [7]. Despite being less densely populated, TB prevalence in rural areas generally rivals that in large cities, perhaps in part because of poverty, malnutrition, and poor access to care [8–10]. Furthermore, the characteristics of TB transmission and contact tracing in rural and urban areas may differ in important ways. Challenges unique to rural settings, such as long distances between healthcare facilities and villages and poor transportation infrastructure, may make implementation of traditional household contact tracing more difficult. Evidence from a recent study in across 70 districts in Vietnam demonstrates that household contact tracing is effective in identifying more patients with TB compared to passive case finding even in rural settings (which made up nearly half the study area), but this study involved intensive human resource and diagnostic efforts, including the use of TB culture [3]. It therefore remains unclear whether active case finding strategies, including household contact tracing, will result in increased case detection when implemented as part of routine care or whether resources are better directed to augment case finding within health facilities. The objective of this cluster-randomized controlled trial was therefore to compare a pragmatically implemented TB contact-tracing strategy (a more resource-intensive approach) with facility-based TB screening (the current standard of care) in terms of the number of individuals initiating treatment for TB at the clinic (cluster) level in a largely rural high-burden setting. We hypothesized that contact tracing would result in more patients with TB started on treatment compared to facility-based screening.

## Methods

### Trial design

We conducted an open-label, parallel cluster-randomized controlled trial in two districts in Limpopo Province, South Africa, comparing two active TB case finding strategies: facility-based screening and contact tracing. Clusters were eligible public-sector primary care clinics within the study area. Clinics were randomized 1:1 to receive assignment to either arm, stratified by district and three levels of historical TB patient volumes (from the year prior to conducting the intervention). Within the contact-tracing arm, clinics were further randomized 1:1 to receive either household-based or incentive-based contact tracing. The intervention period lasted for 18 months, from July 2016 to January 2018. Analyses comparing the two methods of contact tracing are not included here, as a protocol-specified second crossover study comparing these two arms is still underway, and the principal investigators remain blinded to this comparison.

### Study setting and eligibility criteria

This trial was conducted in Vhembe and Waterberg, two largely rural districts in Limpopo Province, purposively selected because of their low (<50/km^2^) population density. The province has the lowest per capita annual income in South Africa (4,213 USD) [11], and TB prevalence was estimated at 300/100,000 [12]. In order to select clinics for inclusion, we used data on the number of Xpert MTB/RIF tests run per clinic in the year prior to the intervention period, provided by the National Health Laboratory Services, and selected the largest clinics per district in which study inclusion was deemed feasible (including willingness to participate, quality of record keeping, and geographic location), for a total of 56 clinics.

### Randomization and masking

Randomization to either the facility-based screening or contact-tracing arm was conducted within each district and within each historical patient volume stratum. We abstracted monthly numbers of patients diagnosed with TB using Xpert MTB/RIF and subsequently started on treatment in each clinic from a 1-year period preceding the start of the intervention (September 2014 to October 2015) in order to categorize the TB patient volume at each clinic as low (<36 patients/year), medium (37–90 patients/year), or high (>90/year). We restricted this historical analysis to a 1-year period because, at the time, clinics were using paper rather than electronic registers, and many did not reliably maintain registers on-site beyond 1 year. Of the 56 clinics, 36 clinics (18 in Waterberg and 18 in Vhembe) fell in the small historical volume stratum, 18 were moderate to large, and two were large (with 10 large or moderate-to-large clinics in each district). Within each stratum and within each district, an equal number of clinics was assigned to each arm. Clinics in the contact-tracing arm were further randomized in a 1:1 ratio to either incentive-based or household-based contact tracing. The random allocation sequence and assignment generation was done by the study biostatistician (B. A. S. N.). The randomization schedule was securely provided to the study data manager and field coordinator who enrolled each cluster. The study principal investigators (D. W. D. and N. M.) were blinded to the study arm allocations and interim results for the duration of the trial.

### Intervention

The facility-based screening intervention (28 clinics) represented the recommended standard of care in South Africa and comprised screening all those attending the clinic and presenting with TB symptoms using WHO’s recommended four-symptom screen [13], often at point of clinic registration. Those screening positive were referred for sputum collection and Xpert MTB/RIF testing. Prior to the start of the intervention, we surveyed all clinics assigned to this arm about their screening practices and gave a standardized training to clinic staff, referencing the South African Department of Health guidelines for TB screening at healthcare facilities [14].

The contact-tracing intervention arm (28 clinics) comprised two distinct and mutually exclusive strategies: household contact tracing (14 clinics) and incentive-based contact tracing (14 clinics). The starting point of both contact tracing strategies was a TB index patient who had started treatment within the previous 2 months prior to recruitment. All patients with TB were eligible, regardless of mode of diagnosis (e.g., bacteriologically confirmed, radiologic) or site of TB disease (e.g., pulmonary or extrapulmonary). In the household tracing arm, the index patient was approached by study staff and consented for a team to visit his or her household. At the household, all contacts present were offered TB screening using WHO’s four-symptom screen, and those contacts not already on TB treatment had sputum collected for Xpert MTB/RIF testing. The study team made up to two additional visits to contact any additional household members missed at the initial visit. In the incentive-based contact-tracing arm, both index patients and their contacts were given monetary incentives if the contact presented to the clinic for screening. Index patients were approached for enrollment by study staff, and the incentive system was explained. Consenting index patients were given 10 paper vouchers to distribute to close contacts (e.g., household members, friends, or coworkers). The vouchers provided information on when to present to the local clinic for TB screening and included a unique coded identifier for the index patient. Contacts presenting with the voucher to the clinic for screening within 2 months of the vouchers being given to the index patient were reimbursed for transport (30 South African rand [ZAR], equivalent to approximately 2 USD) and also given an incentive of 20 ZAR (roughly 1.50 USD). TB screening followed the standard of care (WHO symptom screen with sputum collection). At the end of the 2-month period, the index patient received 20 ZAR (roughly 1.50 USD) for each contact presenting for screening and 100 ZAR (roughly 7 USD) for each contact who was diagnosed with TB through the screening. All incentives were provided in cash, and index patients were not informed which contacts presented for screening or were diagnosed with TB.

Diagnostic testing followed the standard of care for TB diagnosis in South Africa [14]. Those with symptoms suggestive of TB in all arms were asked to provide a single sputum sample. All sputum samples were dropped off in study clinics and transported to the laboratory by the routine courier system by the South African public-sector laboratory, the National Health Laboratory Services. Xpert MTB/RIF was run on each sample (using the G4 cartridge, except for a 2-week period in two clinics in the facility arm and three in the contact investigation arm, where Xpert Ultra was used), and results were reported back to the clinic (in the case of the facility arm and incentive-based contact tracing) following usual clinic procedures or to the study team (in the case of household-based contact tracing), who informed the individual and referred them to the local clinic for TB treatment. TB treatment is available through virtually all public-sector health facilities in South Africa at no charge.

### Outcome

The primary outcome was the number of individuals initiating TB treatment during the 18-month intervention period, measured at the clinic level. Study staff abstracted data on those initiating treatment at each clinic using a combination of electronic and paper medical records and treatment logs. We included all those starting treatment at each study clinic, regardless of location of diagnosis (at the study clinic or at another facility), clinical site of TB disease (pulmonary or extrapulmonary), age of the patient, and mode of diagnosis (i.e., whether they were diagnosed as a result of the intervention).

### Sample size

We used calculations for cluster-randomized comparisons, assuming a geometric mean “effective number of people at risk for TB” (i.e., individuals who would be evaluated for TB were recommended standard-of-care screening procedures in place) of 1,500 per clinic over an 18-month period, based on data from the National Health Laboratory Services. We assumed that, in the less effective arm, 3% of these individuals would initiate TB treatment for a mean yield of 45 patients per clinic over 18 months. We considered the minimum important difference between arms to be a 25% increase in yield, or 56 patients per clinic in the more effective arm. Assuming a coefficient of variation (k) of 0.25 across clinics, we required 28 clusters per arm to detect this difference with 80% power.

### Statistical methods

We characterized the study clinics and patients with TB started on treatment in each arm using percentages for categorical variables and medians and 25th and 75th percentiles for continuous variables. We also plotted the historical annual TB patient volumes (multiplied by 1.5, reflecting the 18-month study duration) against the study period’s number of patients. The unit of analysis was the total number of people with TB who initiated treatment, and the denominator was number of days in the study (18 months for all clinics except for one, which closed during the study period after 12 months). The primary analysis was based on the facility-level rate ratio, and we first calculated an unadjusted ratio of the treatment initiation rates between the two arms and the corresponding 95% confidence interval (CI) [2, 15]. We then adjusted for any residual confounding by district stratification and the historical annual number of people started on TB following a two-stage approach. The first step of this approach fits a Poisson regression to the facility-level counts and the district and historical volume covariates irrespective of study arm. The residuals ratios, calculated as the ratio of the observed over the expected counts, are then used in the second stage to estimate the between-arm rate ratio and the corresponding 95% CI [15]. We further conducted prespecified subgroup analyses according to district and clinic size (modeled as a binary variable, above or below historical annual volume of 36 TB patients). For the main analysis including all clinics, we also compared the results of the two-stage approach to adjustment for covariates to a random effects Poisson regression with random effects to account for the large heterogeneity in the number of people started on treatment between clinics. We conducted two sensitivity analyses: one around 24 TB patients whose TB registration numbers could not be verified and another that excluded patients who had been diagnosed at a different health facility and then transferred to the study clinic. All statistical analyses were conducted using Stata 13 (Stata Corp, College Station, TX, United States).

### Ethics and registration

This trial was approved by the Human Research Ethics Committee at the University of the Witwatersrand in South Africa (clearance number 150216). The Institutional Review Board at the Johns Hopkins Bloomberg School of Public Health provided authorization to rely on the Human Research Ethics Committee at the University of the Witwatersrand for review and continuing oversight of this trial. All participants (TB index patients and their contacts) provided informed written consent. The trial was registered with ClinicalTrials.gov (NCT02808507) prior to the start of enrollment. The full protocol for this trial can be accessed through the supplemental material (See S1 Text). A Data Safety Monitoring Board (DSMB) was established to oversee the trial. Following a first preenrollment meeting, the DSMB was provided with unblinded interim results at all subsequent meetings.

## Results

### Clinic characteristics

**Fig 1:**
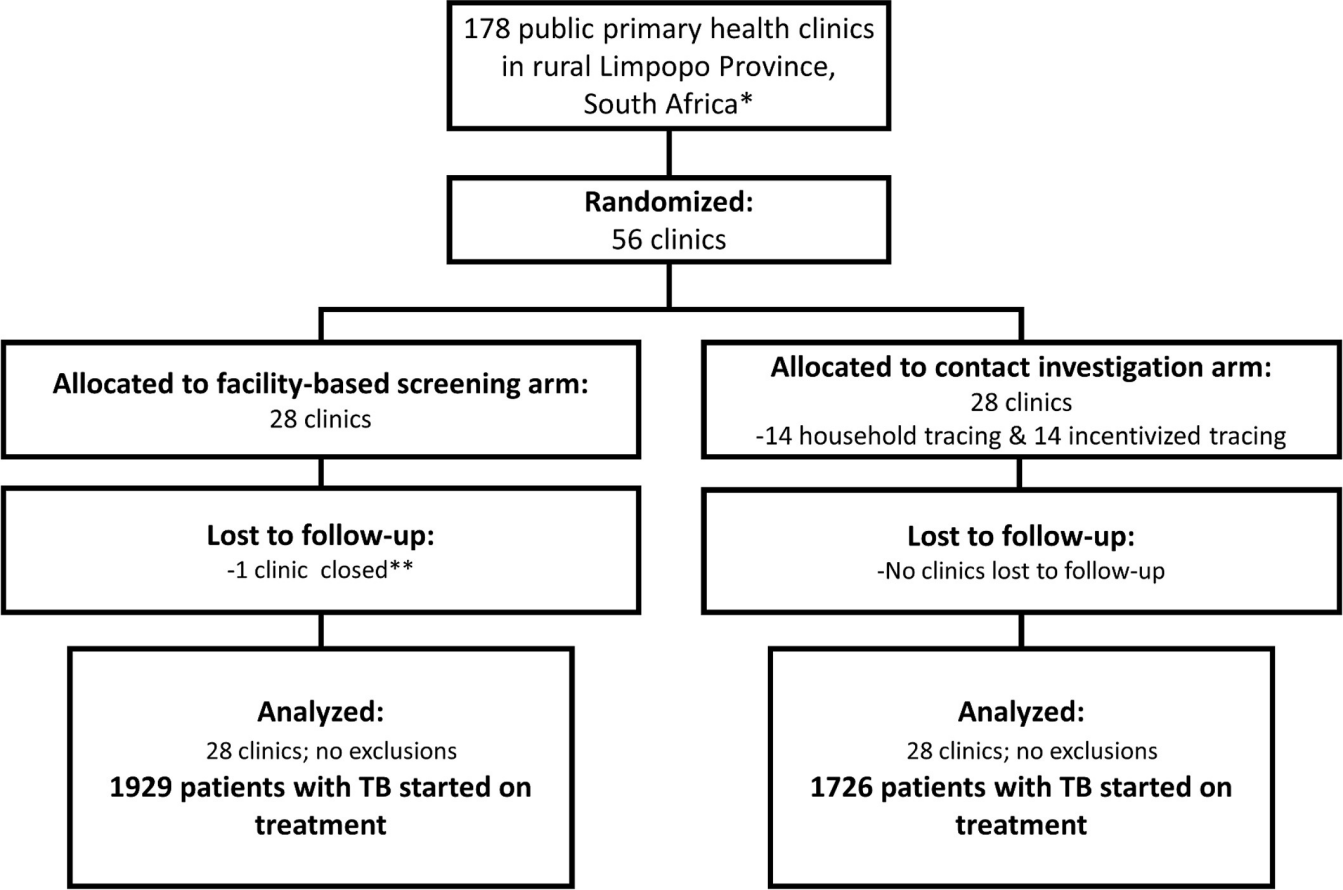
Study CONSORT diagram. *121 clinics in Vhembe district, 57 in Waterberg district. **One clinic closed during the study period and was analyzed with a reduced amount of clinic time accordingly. After clinic closure, TB patients were referred to another study clinic in the facility-based screening arm. TB, tuberculosis.

Fifty-six study clinics were selected out of 178 potential public primary health clinics (PHCs) in the two districts (121 in Vhembe and 57 in Waterberg; Fig 1). Table 1 shows further characteristics of the study clinics. Two types of clinics were included: PHCs provide essential health services only, whereas community health clinics provide primary healthcare services including 24-hour maternity, accident, and emergency care and up to 48 hours of in-patient care. In the contact-tracing arm, 89% were PHCs compared to 64% in the facility-based screening arm (*p*-value for difference 0.055). Eighteen (64%) clinics in the facility-based screening arm and 22 (79%) in the contact-tracing arm were located in rural areas.

**Table 1:** Facility-level characteristics.

Characteristic	Facility-based screening arm	Contact-tracing arm
Number of facilities	28	28
Facility type, *n* (%)^a^		
Community health clinic	10 (35.7)	3 (10.7)
Primary health clinic	18 (64.3)	25 (89.3)
Facility location, *n* (%)		
Rural	18 (64.3)	22 (78.6)
Semi-urban	7 (25.0)	2 (7.1)
Urban	3 (10.1)	4 (14.3)

^a^*p*-Value exact test for independence = .

### Primary outcome

During the intervention period (July 18, 2016–January 17, 2018), there were 1,929 registered TB patients in the facility-based screening arm and 1,726 in the contact-tracing arm (Table 2). A total of 12 new patients were identified by contact tracing. The arms were similar with respect to the characteristics of the participants, which include age, sex, proportion of people living with HIV, and the proportion of patients that were transferred in from other facilities (Table 1).

**Table 2:** Sociodemographic characteristics of patients with TB started on treatment within clinics.

Characteristic	Facility-based screening arm	Contact-tracing arm
Total patients started on treatment (main outcome)	1,929	1,726
Number (%) of patients referred to facility (i.e., transfers)^a^	1,131 (58.7)	988 (57.2)
Median age (lower quartile, upper quartile) of patients, years	38 (28,49)	37 (29,48)
Number (%) of males among patients^b^	1,135 (58.9)	1,001 (57.9)
Age categories, years (%)		
≤5	64 (3.3)	56 (3.2)
6–20	152 (7.9)	133 (7.7)
21–40	841 (43.7)	841 (48.6)
41–60	675 (35.1)	575 (33.3)
61–92	190 (9.9)	124 (7.2)
Missing	4 (0.2)	0
HIV status		
Positive	1,161 (60.2)	1,082 (62.7)
Negative	562 (29.1)	453 (26.3)
Unknown	205 (10.6)	190 (11.0)
Missing	1 (0.1)	1 (0.1)

^a^Total 5 with unknown transfer status.

^b^1 missing sex.

**Fig 2:**
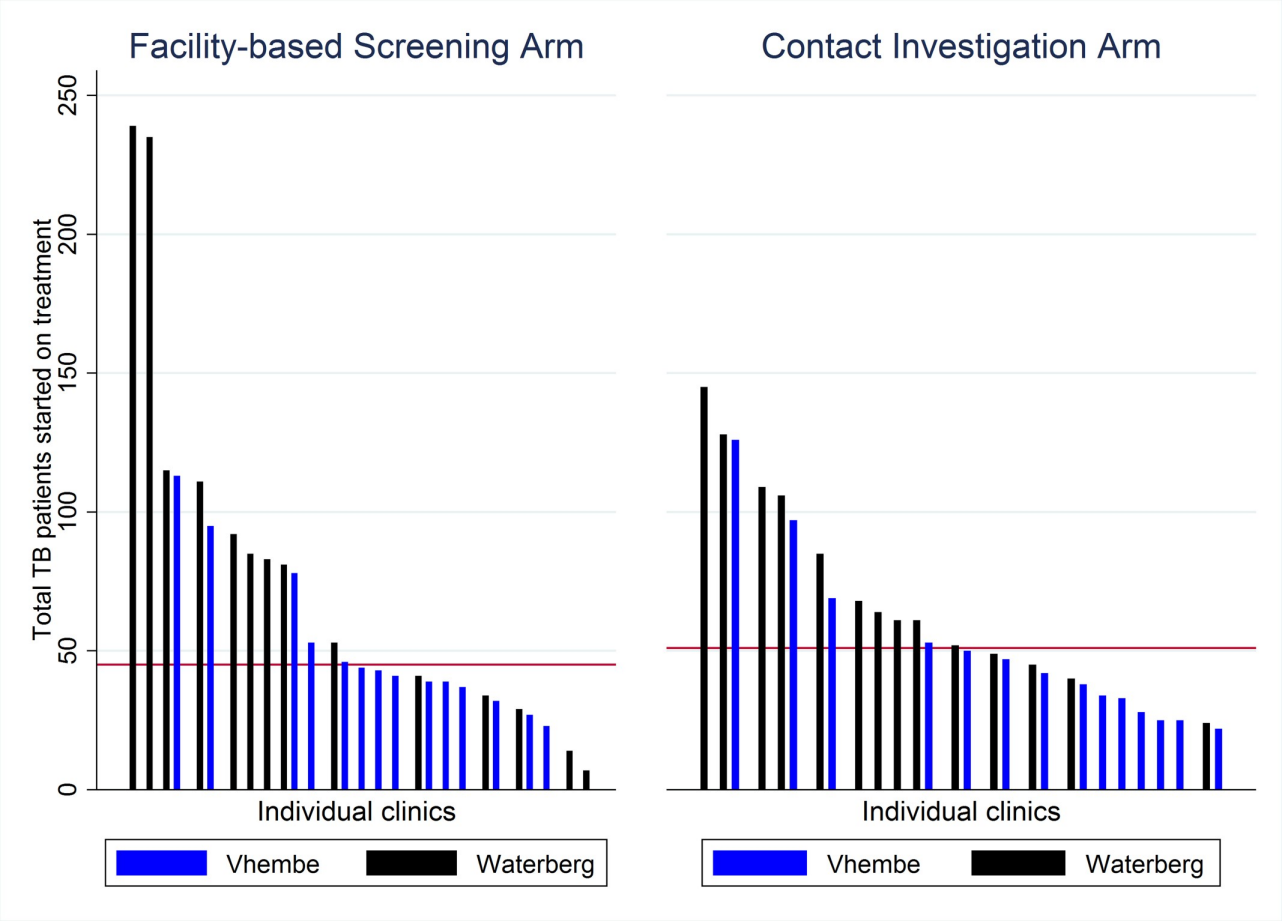
Number of people initiating treatment for TB, by study clinic. Bars represent the number of individuals initiating TB treatment in each study clinic over the 18-month study period and are ordered by volume from largest to smallest. Blue bars indicate clinics in the Vhembe district, and black bars indicate clinics in the Waterberg district, with each panel corresponding to a different study arm. The red horizontal line indicates the median number of people starting treatment in each arm (facility-based screening = 45, contact tracing = 51). TB, tuberculosis.

**Fig 3:**
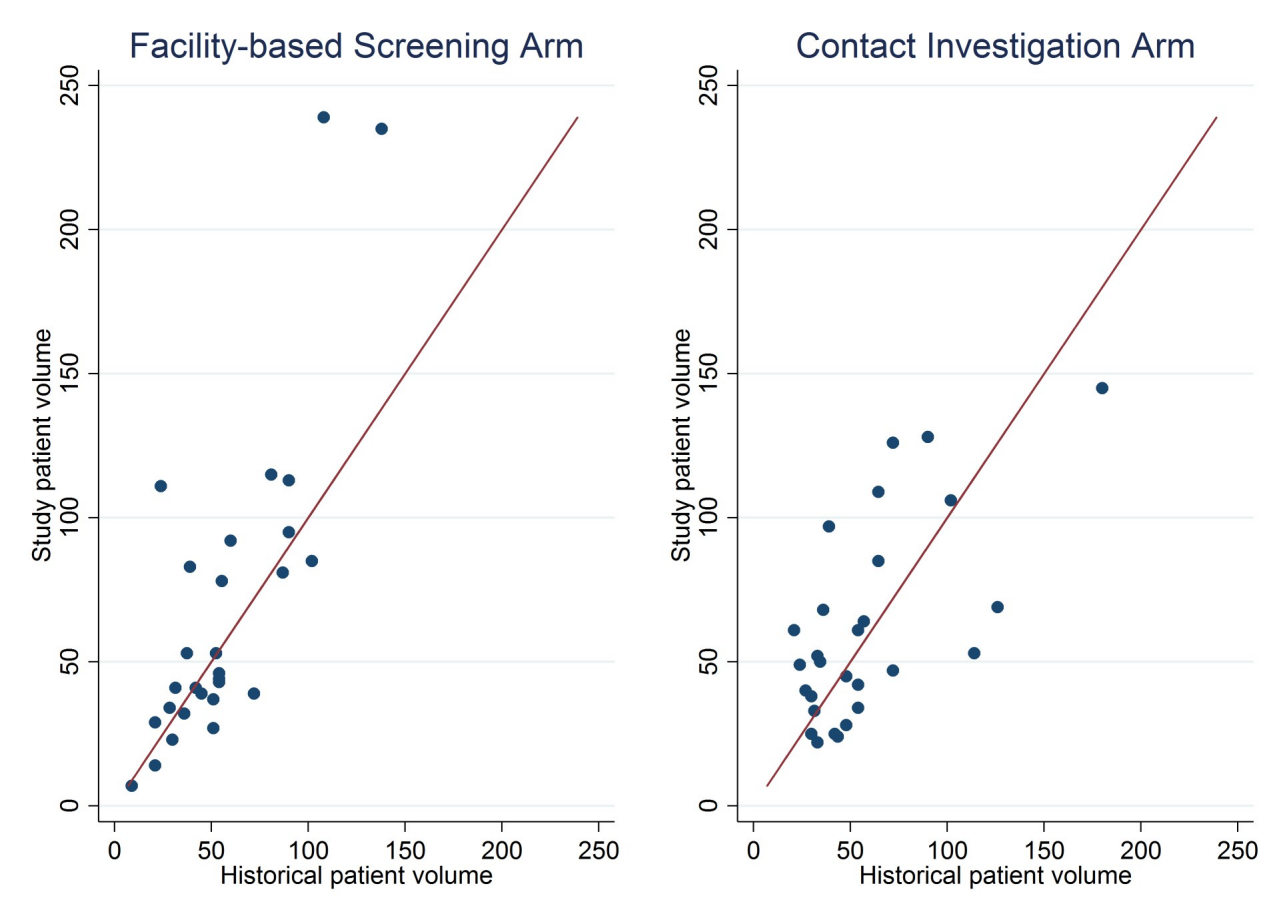
Number of patients with TB started on treatment per clinic, compared against historical annual volumes. Each dot in this graph corresponds to one clinic, with the y-axis showing the number of individuals initiating TB treatment during the 18-month study period and the x-axis showing the number of individuals initiating TB treatment in a 12-month historical period, multiplied by 1.5 for comparison. The diagonal red line has a slope of one and intercept of zero, corresponding to a TB treatment initiation rate that is the same during both periods. TB, tuberculosis.

Clinic patient volumes were distributed evenly across districts and arms, with the exception of two large clinics in the Waterberg district in the facility-based screening arm (Fig 2). Unlike most other clinics in the study, these two clinics had substantially greater numbers of TB treatment initiations during the study period than during a 1-year historical control period (Fig 3). Specifically, one of these clinics had a historical annual volume of 92 patients (anticipated 18-month volume of 138) and had 235 individuals start TB treatment during the study period; the second one had 72 historical treatment initiations (anticipated 18-month volume of 108) and 239 during the study period. In contrast, in the contact-tracing arm, the largest-volume clinic during the prestudy period had 120 patients (anticipated 18-month volume 180) but registered 145 patients during the study period.

**Fig 4:**
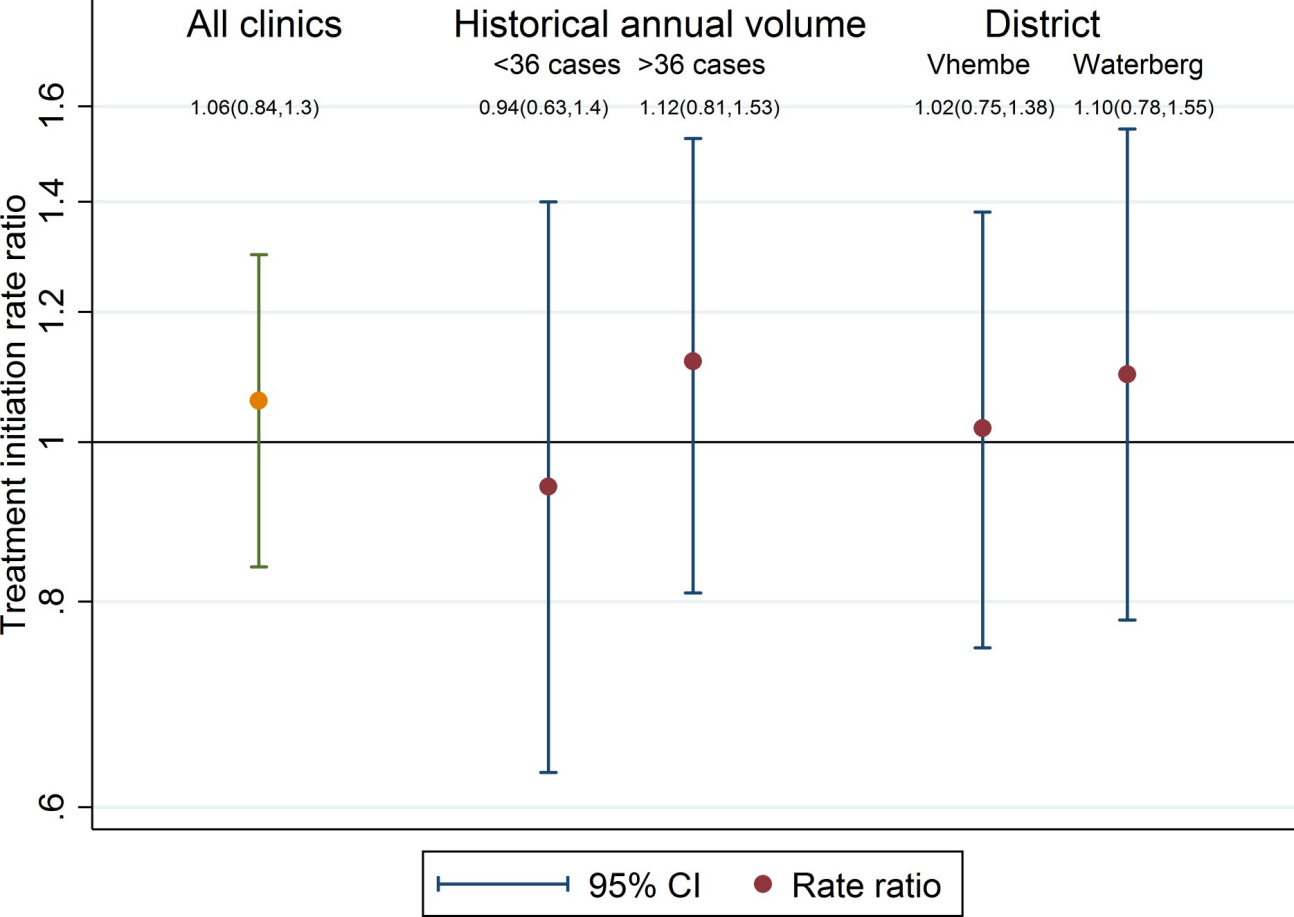
Treatment initiation ratios, comparing the facility-based to the contact-tracing arm. Shown are point estimates (dots) and 95% CIs (error bars) for the treatment initiation ratio, both for the study as a whole (left) and within prespecified subgroups of clinic size (center) and district (right). CI, confidence interval.

The primary analysis, adjusting for balancing factors of district and prior clinic volumes, revealed a treatment initiation ratio of 1.04 (95% CI 0.80–1.3; *p* = 0.73), comparing the facility-based arm to the contact-tracing arm (Fig 4). At an individual (unadjusted) level, the facility-based arm started 11% more patients on treatment than the contact-tracing arm (rate ratio of 1.12 [95% CI 0.75–1.49; *p* = 0.56]), less than the 25% difference considered to be clinically important.

Prespecified subgroup analyses by historical annual volumes and by district showed similar results, with estimated treatment initiation ratios of 0.96 (95% CI 0.64–1.27; *p* = 0.78) among historically smaller clinics, 1.23 (95% CI 0.87–1.59; *p* = 0.29) among historically larger clinics, 1.02 (95% CI 0.66–1.37; *p* = 0.93) in the Vhembe district, and 1.08 (95% CI 0.74–1.42; *p* = 0.68) in the Waterberg district (Fig 4). The estimated treatment initiation ratio was unchanged in sensitivity analyses excluding 24 records whose TB registration numbers could not be verified (1.03, 95% CI 0.82–1.29; *p* = 0.78) and excluding transfers-in (1.02, 95% CI 0.80–1.29; *p* = 0.71).

In the contact-tracing arm, 1,677 contacts from 789 TB index patients were screened for TB (median 1 contact/index patient, lower quartile 0, upper quartile 3). Among the 1,677 contacts, 681 (41%) were symptomatic, among whom 488 (72%) provided a sputum sample for Xpert testing. An additional 193 sputa were collected from nonsymptomatic contacts for a total of 681 contacts tested using Xpert. Twelve contacts (1.8%) tested positive for TB using Xpert. In the facility-based screening arm, among the 1,929 patients with TB started on treatment, 1,427 (74%) were screened with Xpert, and 1,060 (74%) of those screened tested positive.

## Discussion

This pragmatic cluster-randomized trial of 3,755 individuals initiating TB treatment in 56 predominantly rural South African clinics found no difference in treatment initiation rate at the clinic level, comparing the current standard of care (facility-based screening) against feasibly delivered contact tracing based on symptom screening and Xpert MTB/RIF testing. This finding was primarily driven by a lower-than-expected yield of contact tracing, which found only 12 patients across 28 clinics during an 18-month time period. These findings suggest that although close contacts are a high-risk group (estimated TB prevalence 720 per 100,000) and should continue to be prioritized for case finding, the prevalence of TB among contacts may not be higher than that of individuals presenting to healthcare facilities. Diagnostic algorithms with greater sensitivity than symptom-driven Xpert MTB/RIF testing may be necessary for contact tracing to increase TB treatment initiation rates at the clinic level.

The results of this study have important programmatic implications. On one hand, this trial supports the findings of numerous studies that have found a high prevalence of TB among close contacts of index patients [2, 3, 16]. On the other hand, despite this higher-than-background prevalence, the yield of contact tracing in this pragmatic trial was substantially lower than expected. For example, a study of household contact tracing in another rural district of South Africa found a TB prevalence of 6,075 per 100,000, nearly twice as high as the prevalence in this trial [16]. Similarly, the ACT3 trial in Vietnam found a prevalence of 1,788 per 100,000 using intensive contact tracing in 70 districts [3]. TB incidence within South Africa has declined in recent years, falling by about 25% between 2015 and 2017 [17]; however, this trend should have affected both arms equally. The most likely explanation for the discrepancy in findings between these previous studies and the current trial is the diagnostic algorithm; whereas these other studies used TB culture regardless of symptoms, we intentionally adopted a more pragmatically feasible strategy of symptom screening followed by Xpert MTB/RIF. In support of this notion, a preliminary study performed in the same study area as the current trial found a TB prevalence of 3,900 per 100,000, but over 90% of new patients identified were smear-negative and culture-positive (Xpert MTB/RIF was not performed in that study) [18]. Taken together, these studies suggest that contact tracing identifies a population with higher-than-average TB risk, but to increase TB treatment initiation at the clinic level, diagnostic algorithms using highly sensitive diagnostic tests and including asymptomatic individuals will be required.

The contact investigation arm in this study represents a mixture of two strategies: traditional household-based contact tracing (in which study teams visited the households of newly diagnosed TB patients) and incentive-based tracing (in which new TB patients were given small monetary incentives to refer their close contacts to a clinic for screening). It is therefore possible that the observed lack of difference between the facility-based screening arm and contact-tracing arm reflects a substantial effect due to one contact-tracing strategy, counterbalanced by a negative effect from the other strategy. It is also true that the number of contacts screened per index patient in this study (2.1) was relatively low; this reflects, in part, pragmatic decisions to investigate all newly diagnosed TB patients (rather than limiting to bacteriologically confirmed), to perform a single round of contact investigation (up to three visits to the household), and to use a small number of teams to conduct contact investigation across a large number of geographically dispersed clinics. Given the low yield of contact tracing in the study overall coupled with the improbability of a strong negative effect from contact tracing relative to the standard of care, however, this explanation for our results is improbable.

This study has several limitations. First, we chose the number of TB patients started on treatment per cluster (clinic) as the primary outcome, rather than TB incidence per se. This was for reasons of feasibility—there are no reliable estimates of a population denominator for each clinic catchment area from which to estimate incidence. It is therefore possible that our estimates of effect are biased as a result of changing denominators at the clinic level from the historical period to the study period or differences between lab records (used for historical estimates) and clinic treatment records (used during the study period). It is worth noting, however, that study contact-tracing activities were responsible for fewer than 1% of all patients initiating treatment in that arm—making it very unlikely that this explanation alone accounts for our null findings. Second, as discussed above, the contact-tracing arm comprises a mixture of two strategies, and we treated this arm as a single intervention arm for this analysis. Third, the study was single blinded, in which investigators but not the clinics themselves were blinded to intervention assignment, and the 56 study clinics were purposively chosen based on historical TB patient volume from among all possible clinics within the two districts. Finally, this study was conducted in South Africa, an upper-middle-income country with good laboratory and transportation infrastructure, and results may not be generalizable to other settings with more constrained resources.

In conclusion, we provide randomized evidence that contact tracing, implemented pragmatically using an algorithm of symptom screening followed by Xpert MTB/RIF testing, did not result in a difference in the number of TB treatment initiations compared to facility-based screening, the current standard of care. Although contact tracing identifies a high-risk population that should be prioritized for additional case detection by national TB programs, more sensitive diagnostic algorithms are necessary to increase TB treatment initiation at the population level.

## Supporting information

S1 Text Study protocol. This file contains the study protocol.
(PDF)

S2 Text Participant questionnaires. This file contains the questionnaires administered to all participants: TB index patients and their close contacts. TB, tuberculosis.
(PDF)

S3 Text CONSORT checklist. This file contains the CONSORT checklist indicating where in the manuscript each CONSORT element is located.
(DOCX)

S1 Data Study dataset. This file contains the data necessary to reproduce the findings contained within this manuscript.
(XLS)

## Abbreviations

CI: confidence interval
DSMB: Data Safety Monitoring Board
PHC: primary health clinic
TB: tuberculosis
