# Supplementary material for: Contact tracing versus facility-based screening for active TB case finding in rural South Africa: A pragmatic cluster-randomized trial (Kharitode TB)
Source: PLoS Med. 2019 Apr 30;16(4):e1002796. doi: 10.1371/journal.pmed.1002796 (PMC6490908; doi:10.1371/journal.pmed.1002796)
Supplement: S1 Text — This file contains the study protocol. (PDF) [file pmed.1002796.s001.pdf]

COMPARATIVE EFFECTIVENESS/IMPLEMENTATION OF TUBERCULOSIS CASE FINDING IN RURAL SOUTH AFRICA

***DMID Protocol Number:**
15-0005

Sponsored by:
National Institute of Allergy and Infectious Diseases (NIAID)

DMID Funding Mechanism:
R01 AI116787

Industrial Support Provided by:
N/A

Principal Investigator:
David W. Dowdy

DMID Clinical Project Manager
Robin M. Mason

Draft or Version Number:
4.0

31 July 2018

Statement of Compliance

The study will be carried out in accordance with Good Clinical Practice (GCP) as required by the following:

- U.S. Code of Federal Regulations applicable to clinical studies (45 CFR 46)
- ICH GCP E6
- Completion of Human Subjects Protection Training
- NIH Clinical Terms of Award

Refer to: http://www.hhs.gov/ohrp/humansubjects/guidance/45cfr46.htm#46.
http://www.fda.gov/cder/guidance/959fnl.pdf
http://grants.nih.gov/grants/guide/notice-files/NOT-OD-01-061.html
http://cme.cancer.gov/c01/

SIGNATURE PAGE

The signature below constitutes the approval of this protocol and the attachments, and provides the necessary assurances that this trial will be conducted according to all stipulations of the protocol, including all statements regarding confidentiality, and according to local legal and regulatory requirements and applicable US federal regulations and ICH guidelines.

Site Investigator: David Dowdy

Signed: ______________________ Date: July 31, 2018

David Dowdy, MD, PhD
Assistant Professor
Department of Epidemiology
Johns Hopkins Bloomberg School of Public Health

Site Investigator: Neil Martinson

Date: July 31, 2018

Signed: ______________________

Neil Martinson, MBBCh, MPH
Chief Executive Director
Perinatal HIV Research Unit (PHRU)

Table of Contents

page
Statement of Compliance i
Signature Page ii
List of Abbreviations v
Protocol Summary vi

1 Key Roles 8
2 Background Information and Scientific Rationale 10
2.1 Background Information 10
2.2 Rationale 13
2.3 Potential Risks and Benefits 14
2.3.1 Potential Risks 14
2.3.2 Known Potential Benefits 14
3 Objectives 15
4 Study Design 16
5 Study Population 17
5.1 Selection of the Study Population 17
6 Study Procedures/Evaluations 20
6.1 Study Procedures 20
6.2 Laboratory Evaluations 23
6.2.1 Laboratory Evaluations/Assays 23
6.2.2 Special Assays or Procedures 24
6.2.3 Specimen Collection, Preparation, Handling and Shipping 24
7 Study Schedule 25
7.1 Screening and Enrollment 25
7.2 Enrollment/Baseline, if applicable 26
7.3 Follow-up and Final Visits, if applicable 26
7.4 Early Termination Visit, if applicable 27
7.5 Criteria for Discontinuation or Withdrawal of a Subject (or a Cohort), if applicable 27
8 Assessment of Outcome Measures 28
8.1 Specification of the Appropriate Outcome Measures 28
8.1.1 Primary Outcome Measures 28
8.1.2 Secondary Outcome Measures 28
9 Safety assessment and reporting 30
9.1 Definition of Adverse Event (AE) 30
9.2 Definition of Serious Adverse Event (SAE) 30
9.3 Reporting Procedures 30
9.3.1 Serious Adverse Event Detection and Reporting 30
9.3.2 Reporting of Pregnancy 30

Table of Contents - *continued*

page

9.3.3 Procedures to be Followed in the Event of Abnormal Laboratory Test Values or Abnormal Clinical Findings 30
9.3.4 Type and Duration of the Follow-up of Subjects After Adverse Events 31
9.4 Safety Oversight 31
10 Clinical Monitoring Structure 33
11 Statistical Considerations 34
11.1 Study Outcome Measures 34
11.2 Sample Size Considerations 36
11.3 Participant Enrollment and Follow-Up 36
11.4 Analysis Plan 36
11.5 Unblinding 38
12 Access to Source Data/Documents 40
13 Quality Control and Quality Assurance 41
14 Ethics/Protection of Human Subjects 42
14.1 Declaration of Helsinki 42
14.2 Institutional Review Board 42
14.3 Informed Consent Process 42
14.3.1 Informed Consent/Assent Process (in Case of a Minor or others unable to consent for themselves) 44
14.4 Exclusion of Women, Minorities, and Children (Special Populations) 44
14.5 Subject Confidentiality 44
14.6 Future Use of Stored Specimens 45
15 Data Handling and Record Keeping 46
15.1 Data Management Responsibilities 46
15.2 Data Capture Methods 46
15.3 Types of Data 46
15.4 Timing/Reports 46
15.5 Study Records Retention 47
15.6 Protocol Deviations 47
16 Publication Policy 48
17 Literature References 49

SUPPLEMENTS/APPENDICES
A: Study Schedule

List of Abbreviations

ACF	Active Case Finding
AE	Adverse Event
CFR	Code of Federal Regulations
CIOMS	Council for International Organizations of Medical Sciences
CRF	Case Report Form
DMID	Division of Microbiology and Infectious Diseases, NIAID, NIH, DHHS
DSMB	Data and Safety Monitoring Board
FDA	Food and Drug Administration
FWA	Federal-Wide Assurance
GCP	Good Clinical Practice
HIV	Human immunodeficiency virus
ICF	Informed Consent Form
ICH	International Conference on Harmonisation
IEC	Independent or Institutional Ethics Committee
IRB	Institutional Review Board
ISM	Independent Safety Monitor
JAMA	Journal of the American Medical Association
MOP	Manual of Procedures
N	Number (typically refers to subjects)
NEJM	New England Journal of Medicine
NIAID	National Institute of Allergy and Infectious Diseases, NIH, DHHS
NIH	National Institutes of Health
OCRA	Office of Clinical Research Affairs, DMID, NIAID, NIH, DHHS
OHRP	Office for Human Research Protections
ORA	Office of Regulatory Affairs, DMID, NIAID, NIH, DHHS
PI	Principal Investigator
SAE	Serious Adverse Event
SMC	Safety Monitoring Committee
SOP	Standard Operating Procedure
TB	Tuberculosis
WHO	World Health Organization

Title: **Comparative Effectiveness**/Implementation of **Tuberculosis Case Finding** in **Rural South Africa**

Population: 124, 655 male and female participants age 0-99 years including health care workers, tuberculosis patients, household and close contacts of TB patients and attendees of public primary health clinics in Limpopo province, South Africa

SAMPLE

Number of Sites: See Section 1/Appendix

Study Duration: 4 years

Subject Duration: Variable by subject- up to 3.5 years

Objectives:

Primary:

- To measure the comparative effectiveness of active TB case finding in a rural high-burden setting
    a. Measure the comparative yield (number of incident TB cases diagnosed and started on treatment) by facility-based screening versus contact investigation (primary outcome)
    b. Measure the comparative yield of household versus incentive-based contact investigation
    c. Describe the characteristics of individuals screened (and testing positive) under each approach
    d. Link empirical data to a mathematical model to project the likely population-level impact of each case-finding intervention as feasibly implemented (alone, or in combination with other strategies)

Secondary:

- To evaluate the acceptability and adoption of active TB case finding strategies in rural South Africa
    a. Use qualitative methods including focus group discussions and in-depth interviews to determine the relative acceptability of facility-based screening, household contact tracing, and incentive-based tracing
    b. Describe differences in the demographics and symptoms of those who accept versus decline screening
- To describe the comparative implementation of facility-based screening and contact investigation
    a. Measure the reach (coverage and representativeness of individuals screened) of each intervention
    b. Identify barriers to the implementation and maintenance of each intervention

Appendix A: Schedule of Procedures/Evaluations - *continued*

Overall Study TImeline:

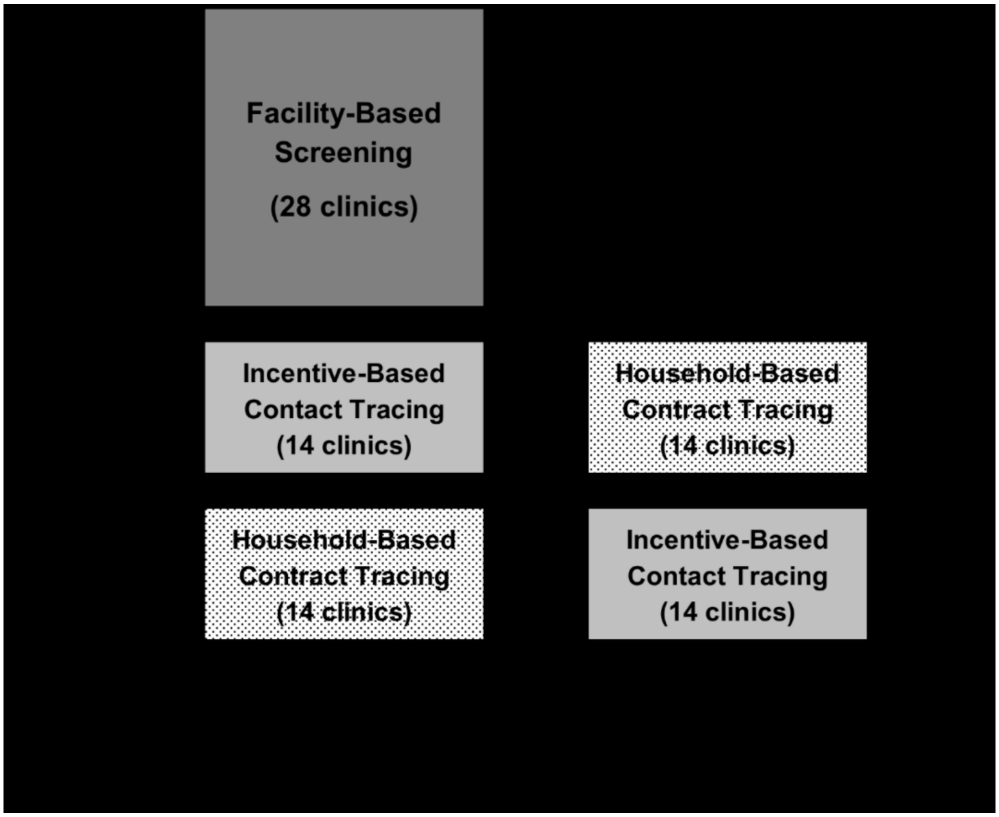

Appendix A: Schedule of Procedures/Evaluations - *continued*

1 KEY ROLES

For questions regarding this protocol, contact Robin Mason (rmason@niaid.nih.gov)

Individuals:

DMID Program Officer/Clinical Project Manager
Robin Mason, MS, MBA
Respiratory Disease Branch/DMID/NIAID
5601 Fishers Lane, Room 8E34
Rockville, MD 20852-9825
Phone: (240) 627-3310
E-mail: rmason@niaid.nih.gov

DMID Medical Officer
Francisco J. Leyva MD, PhD, ScM
TB, Leprosy and other Mycobacterial Diseases Section
Respiratory Diseases Branch/DMID/NIAID
5601 Fishers Lane, Room 8E27
Rockville MD 20852-9825
Phone: (240) 627-3347
E-mail: francisco.leyva@nih.gov

Principal Investigator: David Dowdy, MD PhD
Assistant Professor
Johns Hopkins Bloomberg School of Public Health
615 N. Wolfe St., E6531
(410) 614-5022 (phone), (410) 614-0902 (fax)
ddowdy1@jhmi.edu

Local (South Africa) Principal Investigator: Neil Martinson, MBBCh DCH MFGP MPH
Chief Executive Director
Perinatal HIV Research Unit
Chris Hani Baragwanath Hospital
Soweto, South Africa
+27 11-989-9762 (phone)

Appendix A: Schedule of Procedures/Evaluations - *continued*

martinson@phru.co.za

Institutions:

Johns Hopkins Bloomberg School of Public Health
615 N Wolfe St, Baltimore, MD 21205 USA
David Dowdy, MD, PhD
410-614-5022 (phone)
410-614-0902 (fax)
ddowdy1@jhmi.edu

Perinatal HIV Research Unit
Chris Hani Baragwanath Hospital, Soweto, South Africa
Neil Martinson, MBBCH MPH
+27 11-989-9762 (phone)
+27 11-989-9762 (fax)
martinson@phru.co.za

Appendix A: Schedule of Procedures/Evaluations - *continued*

2 BACKGROUND INFORMATION AND SCIENTIFIC RATIONALE

2.1 Background Information

Tuberculosis (TB) remains a global health emergency, responsible for 8.6 million new cases and 1.3 million deaths in 2012.[1] Worldwide, TB incidence has been declining since 2004 at a rate of only 2% per year.[1] The global community has recognized that this rate of progress – with declines in incidence nearly matched by population growth – is unacceptable for a curable disease, and the Stop TB Partnership has set very ambitious targets that were approved by the World Health Assembly in May 2014. These targets include a 50% reduction in TB incidence from 2015 to 2025, and ending the global TB epidemic by 2035 through a 95% reduction in TB deaths.[2] To meet these ambitious targets, equally ambitious control efforts will be required – and to have impact by 2025, such efforts must be implemented today. The top TB control priority of the World Health Organization (WHO) is to "find the missing 3 million" – the 3 million individuals who develop infectious TB each year but are either never diagnosed as having TB, or diagnosed but not notified to public health authorities.[3] In South Africa, where the vast majority of TB cases receiving appropriate TB treatment are notified, these "missing" individuals are comprised primarily of those who are never diagnosed. Furthermore, those who are diagnosed often receive that diagnosis only after a long period of infectiousness ending in severe symptoms. Rapid diagnosis and initiation of treatment is a universally effective strategy for reducing the transmission of infectious diseases, from respiratory infections (e.g., influenza) to sexually transmitted infections to vector-borne diseases; it is essential that we adopt a similar strategy for TB control. Unless efforts are made to actively find and treat those with undiagnosed, infectious TB in the community, global targets for TB control will not be met, regardless of any future innovations in diagnostic testing, treatment, or prevention.

Mathematical models consistently conclude that, in the absence of a technological breakthrough (e.g., a new vaccine), active TB case finding is the intervention most likely to substantially reduce TB incidence. Two models independently estimated that periodic waves of community-based active case finding (ACF) could reduce TB incidence by 22-33%, albeit in generic "context-free" populations.[4, 5] In an agent-based simulation model, we found that household contact tracing, if fully implemented, could effect a 10-15% reduction in TB incidence.[6] Another model from our group, using data from a demonstration project in urban Karachi,[7] showed that even a one-year ACF campaign could reduce TB incidence by 20% in the long term.[8] In short, although we lack randomized empirical evidence that any TB-specific intervention (other than isoniazid preventive therapy and BCG for childhood TB) reduces TB incidence at the population

Appendix A: Schedule of Procedures/Evaluations - *continued*

level, we cannot afford to wait decades for the next technological breakthrough or large cluster-randomized trial. Active TB case finding is not only good for patients; it is also the intervention with the strongest scientific evidence of the ability to reduce TB incidence to a substantial degree over the next 10 years.

There are two major reasons that active case finding for TB has not been more widely adopted, as it has in "test and treat" strategies for HIV, malaria, gonorrhea/chlamydia, and many other infections. First is the perceived resource intensity of broader TB testing without the availability of a cheap, point-of-care test; however, in South Africa, our team's modeling suggests that – by averting future cases of disease – ACF efforts that cost even $400 per case detected could save the healthcare system money over a 10-year horizon.[9] The second barrier is the lack of population-level randomized evidence that ACF (i.e., more rapid diagnosis and treatment) reduces incidence.[10, 11] This standard has not impeded successful "test and treat" efforts in other infectious diseases, nor the scale- up of novel diagnostic tests for TB (e.g., Xpert MTB/RIF) that, like ACF, also benefit patients directly by detecting disease at earlier, less symptomatic stages. In short, the question regarding ACF for TB should not be *whether* ACF efforts should be implemented, but rather *how* they should be implemented in a wide range of settings with different population, structural and epidemic characteristics.

<u>The Need for Comparative Effectiveness Research in Active TB Case Finding</u>

Before implementing active TB case finding in any given setting, it is critical to understand which ACF intervention is most likely to have the greatest impact. Although often modeled as a monolithic community-wide effort to find prevalent cases, ACF for TB is more appropriately conceptualized as a wide array of strategies that focus intensive efforts to increase TB detection among selected populations at greatest risk for disease.[12] The most widely implemented ACF approach is screening household and close contacts of newly identified TB cases. Contact tracing is standard practice in most well-resourced, low-burden countries,[13, 14] recommended for all settings by the WHO,[15] and is widely considered essential to maintaining TB rates at the "near-elimination" levels targeted by the World Health Assembly for 2035.[2] Our team conducted a study of household contact tracing in a South African city (Klerksdorp) and found an extremely high prevalence of culture-proven TB among contacts (6.1%).[16] The majority of these cases had no symptoms, meaning that they could be treated before TB morbidity – and likely the majority of transmission – ensued. However, although household contact tracing can identify people with early TB, it may not be the most effective or efficient method for doing so.

Alternative ACF strategies may target broader populations than just contacts of active cases. For example, DETECTB, set in urban Zimbabwe, examined mobile van and door-to-door community ACF approaches for TB, and found a 40% decrease in TB prevalence after a two year campaign.[17] ZAMSTAR, set in South Africa and Zambia, employed household contact screening, and resulted in an 18% reduction in TB prevalence and a 45% reduction in TB incidence over 3 years, though neither finding was statistically significant ($p = 0.06$).[10] Other

Appendix A: Schedule of Procedures/Evaluations - *continued*

smaller studies have suggested that a substantial number of TB cases can be found simply by screening individuals who present to healthcare facilities for any reason (not just for TB symptoms).[18, 19] Ultimately, the ideal approach to active TB case-finding – whether tracing contacts or screening a broader population – remains uncertain, and is likely to vary by setting. If we are to make serious progress toward reaching global TB control targets, therefore, we need to understand which types of case-finding interventions are likely to be the most effective, as actually implemented in key settings.

One key setting that is particularly neglected in discussions of ACF for TB is the rural, high-burden region. In sub-Saharan Africa, 60% of the population still lives in rural areas,[20] and migration from rural to urban settings may represent a key driving force in TB dynamics.[21] Despite being less crowded, TB prevalence in rural areas generally rivals that in large cities, perhaps in part because of poverty, malnutrition, and poor access to care.[22-24] Furthermore, the sites of TB transmission may be very different in rural settings than in urban ones. In the urban setting, public transit may be one important site, whereas households may only account for one-fifth of transmission,[25, 26] whereas the household may be more important in rural communities with large family compounds and fewer social venues. Resource availability and patient economic burden may also differ dramatically between urban and rural settings. As such, ACF interventions that are most effective in cities (where the majority of TB research occurs) may not generalize to the majority of the population in sub-Saharan Africa. If we are to achieve reductions in TB incidence and prevalence for *all* people in high-burden settings, we must understand which TB control interventions are most likely to be feasible and effective in rural settings as well as in urban ones.

Comparative Implementation and Effectiveness Study of Active TB Case Finding

Here, we propose to evaluate, in randomized fashion, the comparative acceptability, implementation, effectiveness, and cost-effectiveness of different strategies for active TB case finding in 56 public clinics in rural South Africa (Figure 3). Our proposed research sites are Vhembe and Waterberg, two rural districts in Limpopo province with a TB incidence (350 per 100,000/year) that is nearly three times the global average and the site of an ongoing pilot project of household contact investigation. Limpopo borders Zimbabwe to the north and (as in many rural African districts) is home to many people who migrate to cities for seasonal work. We chose two approaches of particular relevance to the rural African setting as our primary interventions. First, contact investigation takes advantage of the fact that a preponderance of TB transmission from index cases in rural settings may take place among people whom the index case actually knows. Second, facility-based screening capitalizes on the fact that most individuals who are eventually diagnosed with active TB have contacted the healthcare system in the months immediately preceding their diagnosis,[27] and that healthcare facilities in rural settings are generally more closely linked to the public sector and easier to enumerate. Thus, contact investigation offers "depth" in that it accesses individuals who may not come to the healthcare system on their own[28] – an important dynamic in rural settings. By contrast, the

Appendix A: Schedule of Procedures/Evaluations - *continued*

facility-based approach provides "breadth" in that all individuals, not just those with some relationship to TB cases, are eligible for screening.

Importantly, the success or failure of active TB case finding – particularly in rural settings – depends not only on efficacy as evaluated in a trial setting, but also on real-world implementation and effectiveness. It is therefore essential to evaluate not only the comparative ability of ACF strategies to identify TB cases in an idealized setting, but also the comparative acceptability, implementation feasibility, and cost-effectiveness of these approaches. Our pilot project in Vhembe has demonstrated that household contact tracing can be feasibly implemented in this setting, and that it results in a high yield of active TB cases. However, it remains unclear whether contact tracing would be more acceptable, feasible, effective, or cost-effective than facility-based screening, nor whether a contact tracing strategy that brings the health system to patients' households is more appropriate than one based on incentives (i.e., conditional transfers of mobile phone airtime). Each of these ACF approaches is not only important in its own right, but also paradigmatic of key sites/mechanisms (i.e., households, healthcare facilities, and patient-focused incentives) that might be leveraged to actively find TB cases in other rural, high-burden settings as well. Thus, a comparative implementation/effectiveness evaluation of contact investigation versus facility-based screening will not only inform the case-finding approach most likely to succeed in Vhembe, but it will provide key insights likely to generalize to other rural settings in sub-Saharan Africa as well.

2.2 Rationale

Our proposed research sites are Vhembe and Waterberg, two rural districts in South Africa with a TB incidence (350 per 100,000/year) that is nearly three times the global average and the site of an ongoing pilot project of household contact investigation. We have chosen these sites as they comprise a well-resourced setting for research, yet are rural enough to stand in for many rural settings in other African settings to which we hope to generalize our research findings. Our chosen study population includes health care workers at public primary care clinics, TB patients identified at these clinics, household and close contacts of TB patients, as well as attendees of these clinics. Health care workers are included in the formative qualitative research as these are the individuals who will primarily be responsible for carrying out the TB case finding strategies we are testing. We include TB patients, their household and close contacts, and attendees of public health clinics as these are the groups who will be targeted by the active case finding strategies we propose.

Appendix A: Schedule of Procedures/Evaluations - *continued*

2.3 Potential Risks and Benefits

2.3.1 Potential Risks

The risks faced by participants in this study relate to consequences due to breach of confidentiality. Disclosure of any clinical information, including HIV and TB status, could be potentially damaging to the study participants. Household visits by the study team may engender stigma by neighbors.

No special risks exist in this study for pregnant women which are above and beyond those for non-pregnant study participants.

We feel these risks are justified in light of the potential benefits to the individuals (described below) as well as the importance of the knowledge gained and future benefits to society. TB is a leading infectious cause of disease and death, particularly in South Africa. Only 2/3 of all cases are diagnosed and notified to begin appropriate treatment. In order to speed up the decline of TB, efforts must be put in place to actively find, diagnose and start these missed cases on treatment. Particularly in rural areas, these efforts are not well implemented, and are viewed and too cumbersome and costly. This study is important as it aims to examine the evaluate not only the comparative ability of three strategies for active TB case finding, but also the comparative acceptability, implementation feasibility, and cost-effectiveness of these approaches.

2.3.2 Known Potential Benefits

The direct benefit from participation in this study is limited to those participants who are tested for TB. In these participants, we may be able to identify TB earlier in the disease course than if they developed symptoms and presented to the healthcare system.

Appendix A: Schedule of Procedures/Evaluations - *continued*

3 OBJECTIVES

1. To evaluate the acceptability and adoption of active TB case finding strategies in rural South Africa
    a. Use qualitative methods including focus group discussions and in-depth interviews to determine the relative acceptability of facility-based screening, household contact tracing, and incentive-based tracing
    b. Describe differences in the demographics and symptoms of those who accept versus decline screening

2. To describe the comparative implementation of facility-based screening and contact investigation
    a. Measure the reach (coverage and representativeness of individuals screened) of each intervention
    b. Identify barriers to the implementation and maintenance of each intervention

3. To measure the comparative effectiveness of active TB case finding in a rural high-burden setting
    a. Measure the comparative yield (number of incident TB cases diagnosed and started on treatment) by facility-based screening versus contact investigation (primary outcome)
    b. Measure the comparative yield of household versus incentive-based contact investigation
    c. Describe the characteristics of individuals screened (and testing positive) under each approach
    d. Link empirical data to a mathematical model to project the likely population-level impact of each case-finding intervention as feasibly implemented (alone, or in combination with other strategies)

4. Evaluate the comparative costs and cost-effectiveness of each case-finding strategy
    a. Measure the incremental cost and cost-effectiveness (incremental cost per new TB case identified and started on treatment) for each case-finding strategy, from the healthcare perspective
    b. Compare the costs and consequences of each approach (including the current standard of passive case detection) for members of the target screening population

Appendix A: Schedule of Procedures/Evaluations - *continued*

4 STUDY DESIGN

We will conduct a cluster-randomized comparative effectiveness study involving 56 clinics in the rural districts of Vhembe and Waterberg, South Africa. We will perform two comparisons: (1) A parallel comparison (28 clinics each) of augmented facility-based TB screening (sputum collection and Xpert MTB/RIF testing of all coughing patients presenting to clinic for any reason) versus contact investigation of active TB cases; and (2) A cluster-crossover evaluation within the contact investigation arm, comparing traditional household contact tracing versus incentive-based tracing (giving airtime coupons in exchange for contact referrals presenting at the clinic). For each intervention, we will measure comparative effectiveness in terms of cases identified as well as the cost-effectiveness and feasibility of implementation. During the lead-up to the intervention period, we will perform qualitative work (in-depth interviews and focus groups) with the target population in order to refine intervention design and ensure acceptability. (See Study Scheme)

Appendix A: Schedule of Procedures/Evaluations - *continued*

5 STUDY POPULATION

5.1 Selection of the Study Population

Sample Size

The total sample size we expect to enroll is 128,171. We make no exclusions based on sex, age or race for enrolment, therefore we expect that our final enrolled study population will be roughly representative of those living within the study area. Based on Vhembe district demographics, we anticipate enrolling 69,212 women (54% of the study population), 43,578 children under 15 years of age (34% of the study population), and 124,325 Black Africans (97% of the study population).

<u>We expect our target sample size to break down as follows:</u>
In-depth interviews: 25
Focus group discussions: 30
Pre-intervention record review: 56,000 (Approximately 1,000 TB suspects/cases per clinic per year)
Index cases in contact-tracing arm: 3,150
Household and close contacts in contact-tracing arm: 9,450
Facility-based participants: 3,516 (1500 Xpert positives and 2016 Xpert negatives)
Washout period record review: 14,000
Post-intervention record review: 42,000

Study Population Sources

Participants will mostly be drawn from the patient population attending the 56 study clinics, as well as the households and close contacts of newly diagnosed TB cases. The only exceptions to this will be during the formative work, when participants in the healthcare worker in-depth interviews will be chosen from staff at several representative clinics from among the 56 clinics, and participants in the community member in depth interviews will be drawn from the catchment area of several representative clinics, recruited through key stakeholders and community organizations.

Appendix A: Schedule of Procedures/Evaluations - *continued*

Eligibility Criteria

Subjects in each of the below participant categories must meet each eligibility criteria for that category:

In-depth interviews- TB cases

- Age 18-99 years
- Informed consent provided
- Newly diagnosed with TB in past 2 months at a study clinic

In-depth interviews-Clinic attendees

- Age 18-99 years
- Informed consent provided
- Attending one of the study clinics for routine care

In-depth interviews-Household members of TB cases

- Age 18-99 years
- Informed consent provided
- Living in same household as a newly diagnosed (last 2 months) TB case from a study clinic

In-depth interviews-Healthcare workers

- Age 18-99 years
- Informed consent provided
- Currently employed as a nurse or physician at a study clinic

Focus groups: TB patients

- Age 18-99 years
- Informed consent provided
- Patient currently on TB treatment at a study clinic

Pre-intervention file review

- Age 0-99 years
- Listed in the TB suspect register or TB treatment register at any of the 56 clinics for the period of April 2015-March 2016.

Facility-based screening arm

- Age 0-99 years
- Informed consent provided (or assent plus parent/guardian consent)
- Attending any of the study 28 study clinics in the facility-based screening arm

Contact tracing arm- Index Case

- Age 0-99 years

Appendix A: Schedule of Procedures/Evaluations - *continued*

- Informed consent provided (or assent plus parent/guardian consent)
- Newly diagnosed (last 2 months) with TB at any of the 28 study clinics in the contact tracing arm

Contact tracing arm- Household Contact

- Age 0-99 years
- Informed consent provided (or assent plus parent/guardian consent)
- Living in the same household as an enrolled Index case (see above)

Contact tracing arm- Non-household Close Contact

- Age 0-99 years
- Informed consent provided (or assent plus parent/guardian consent)
- Referred to the study as a close contact of an enrolled Index case (see above)

Washout period record review

- Age 0-99 years
- Listed in the TB suspect register or TB treatment register at any of the 56 study clinics

Post-intervention period record review

- Age 0-99 years
- Listed in the TB suspect register or TB treatment register at any of the 28 study clinics in the facility-based screening arm

Exclusion Criteria

- Unable to provide informed consent (if required)

Appendix A: Schedule of Procedures/Evaluations - *continued*

6 STUDY PROCEDURES/EVALUATIONS

6.1 Study Procedures

Record Review: Pre-intervention record review

TB suspect logs, lab registers and treatment registers at each of 56 clinics will be reviewed in order to gather the following data elements on all TB suspects and newly diagnosed TB cases (totaling approximately 56,000 subjects): Age, sex, HIV status, ART status, most recent CD4 count, date of evaluation, date of diagnosis and date of treatment initiation.

In-depth interviews

We will conduct a series of in-depth interviews with key informants, including TB health care workers and patients and community members who comprise the target populations of interest, including: (1) index TB patients who would be given coupons; (2) household members of TB patients; (3) community members of high-TB-incidence neighborhoods;(4) individuals presenting to study clinics for routine care; and (5) health care workers involved in TB service provision. We plan to conduct up to 5 interviews for each of these 5 subgroups for a total of 25 subjects. Each semi-structured, in-depth interview will be approximately 45 minutes in length and will cover the following domains: individuals experiences with TB symptoms prior to diagnosis, the TB diagnostic process, clinic experiences and quality of TB care, appropriate reimbursement amounts (for incentive-based tracing) feasibility and acceptability of different methods of ACF in early case detection, perceived benefits (e.g., reduced symptom burden, reduced transmission, confidentiality) and potential unintended consequences (e.g., stigma, inconvenience, over-diagnosis) of active TB case finding methods. In-depth interviews will be conducted by trained qualitative research interviewers using the attached interview guide.

Focus group discussions

Based on initial findings from the IDIs, we will hold a series of focus group discussions among patients undergoing treatment for TB in the study clinics during the pre-intervention period. Focus group discussions will examine group level perceptions of approaches to TB testing in the community, reasons why active TB case finding would or would not be perceived as feasible and acceptable, sources of TB-related stigma, perceptions of TB risk, and willingness to participate in the specific intervention strategies being studied here. We will conduct 3 focus groups with 8-10 individuals per group, for a total of 30 subjects. Whereas the in-depth interviews will focus on individual TB-related views and experiences, focus group discussions will examine group-level shared perceptions and norms surrounding TB and its detection and treatment, as well as specific areas of consensus or divergence regarding how to best

Appendix A: Schedule of Procedures/Evaluations - *continued*

operationalize each of the 3 ACF intervention strategies. Focus group discussions will be conducted by trained qualitative research interviewers using the attached discussion guide. These focus groups will inform our secondary objective of evaluating the acceptability and adoption of the active TB case finding strategies.

Facility-based screening arm
This strategy will be implemented at all clinics (n=28) within this arm for 18 months. Study staff will encourage providers at each of the clinics to screen all consenting patients attending the clinic, regardless of the original reason for clinic presentation. Upon presenting for care (e.g., while waiting for their healthcare provider), patients will be informed about the study and screened for cough of any duration, fever, weight loss, or night sweats. Participants who are symptomatic and provide a sputum specimen (according to the clinic standard of care) will be given a study flyer informing them that they may be contacted by our study staff, and a brief summary of the study. Per standard of care, all sputum samples will be sent to the local National Health Laboratory Service laboratory for Xpert testing.

For all participants tested by Xpert, clinic nurses will ask if they can be contacted by phone in case no study staff are present at the clinic, or will refer the participant to study staff present at the clinic. If contacted by phone, study staff will administer a verbal consent which will be documented, prior to administering the questionnaire. If in person, study staff will administer written informed consent prior to administering the questionnaire. Further details of the consent process can be found in Section 14.3. We estimate enrolling approximately 1,500 Xpert positive subjects in this arm. For participants testing Xpert-negative, clinic staff will rotate among the 28 study clinics and randomly select 1 Xpert negative individual per clinic per week over the study period, for a total of 2,016 Xpert negative subjects). Study staff will consent Xpert negatives as described above for Xpert positives.

The questionnaire for both Xpert-positives and negatives will cover clinical details, TB risk factors, reasons for the clinic visit, care-seeking behaviour, and acceptability of the intervention. The clinic records of the remaining Xpert-negative participants not interviewed in person will be reviewed. All Xpert-negative participants at all 28 study clinics in this arm will be identified through the TB suspect register and laboratory register. Data on each participant not previously interviewed will be abstracted from these registers. The following data elements will be gathered: age, sex, HIV status, ART status, most recent CD4 count, date of evaluation, TB symptoms. The consent process for these individuals is detailed in Section 14.3.

Household contact tracing
This strategy will be implemented for 18 months at half of the clinics within the contact tracing arm (n=14) and, after a six-month washout period, for 18 months in the other half of the clinics in that arm (n = 14). All adults (and guardians for children) with newly diagnosed pulmonary TB

Appendix A: Schedule of Procedures/Evaluations - *continued*

will be approached for enrolment as described above. Consenting patients (estimated as 1,575 subjects) will be asked for a home address (with landmarks), mobile phone number, and convenient time for a household visit. A mobile field team consisting of a nurse and lay health worker (both research staff) will circulate between the 14 primary care clinics, with notifications of all new TB cases made from the clinic to the team by mobile phone. The field team will visit the household of each consenting index case within two weeks of notification; each visit will consist of a household census (including demographic data), consent of all eligible household members for questioning and TB screening, administration of a brief questionnaire (including symptomatology, self-reported HIV and TB history, care-seeking behavior, and acceptability of the intervention), and sputum collection for testing with Xpert MTB/RIF. Each participant will also be offered voluntary HIV counselling and testing. The team will make up to three visits to each household as needed in order to approach all household contacts for study enrolment. We estimate enrolling a total of 4,725 household contact subjects in this arm. Positive TB test results will be conveyed back to the household within a week through a follow-up visit to the household, and newly identified TB cases will be referred for treatment through the public health system. At the conclusion of 18 months of this strategy, clinics will switch to the index case-drive contact referral arm.

<u>Incentive-based contact tracing</u>
This strategy will be implemented in the opposite half of the clinics randomized to contact investigation. All adults (and guardians of children) with newly diagnosed active TB will be identified and approached for enrolment as described above. However, rather than a household visit, each consenting individual (estimated as 1,575 subjects) will be provided with a set of 10 coupons to give to household members and close contacts whom the index case believes may be at greatest risk. These coupons will contain instructions on how to come to the clinic for free TB screening (e.g., when the study team will be at each clinic), and will have an expiration date of 2 months from the time of initial TB diagnosis. When a contact presents at the clinic with the coupon, the contact will receive a small amount of money and a transport allowance based on the distance travelled to the clinic. If the contact is diagnosed with active TB and starts treatment, the contact will receive an additional larger amount of money. Study staff will document which index case the contact is linked to, and whether the contact is diagnosed with active TB. At the end of the 2 month period in which the coupons are valid the index will receive a grocery voucher for the total incentive earned (e.g., total amount for contact(s) who presented for screening + total amount for contact(s) diagnosed with active TB and starting treatment). We estimate enrolling a total of 4,725 household contact subjects in this arm. A study team similar to that in the household contact tracing arm will rotate between clinics on a fixed schedule (described on the coupons); a research nurse will administer the brief demographic and clinical questionnaire (~40 questions) to each contact, perform a TB symptom screen, offer HIV testing, and collect a sputum sample for Xpert MTB/RIF.

Appendix A: Schedule of Procedures/Evaluations - *continued*

Incentive structure for this arm:
Amounts are approximated and based on regional transport costs. Final incentive amounts will be informed through formative qualitative work and submitted to ethics review prior to impleentation of that study arm.

Index case

- Per adult contact presenting for screening: R20 in grocery voucher
- Per any number of paediatric contacts: R20 in grocery voucher
- Per contact diagnosed with TB: R100 in grocery voucher

Contact

- For presenting for TB screening at clinic: R20 plus transport reimbursement
- For initiating TB treatment (if diagnosed): R40 plus transport reimbursement

<u>Washout and post-intervention record review</u>
TB suspect logs, lab registers and treatment registers at each of 56 clinics will be reviewed during the washout period, and thereafter in 28 clinics after the facility-based case finding intervention arm has been completed. The following data elements on all TB suspects and newly diagnosed TB cases will be gathered:
Age, sex, HIV status, ART status, most recent CD4 count, date of evaluation, date of diagnosis, method of diagnosis and date of treatment initiation.
The period of record review: August 2017-January 2018 (Washout period in all 56 clinics), February 2018-May 2019 (Post-intervention in 28 facility-based screening clinics).
During the washout period record review, we estimate 95% will be among TB suspects (13,300 subjects) while 5% will be among TB cases (700 subjects). During the post- intervention record review, we similarly estimate 95% will be among TB suspects (39,900 subjects) while 5% will be among TB cases (2,100 subjects).

6.2 Laboratory Evaluations

6.2.1 Laboratory Evaluations/Assays

<u>Xpert MTB/RIF</u>

Xpert MTB/RIF is a molecular assay that detects the presence of *Mycobacterium tuberculosis* in sputum, as well as reports on sensitivity to the antibiotic rifampicin as an indicator for multidrug resistant tuberculosis. Xpert MTB/RIF assays will be carried out

Appendix A: Schedule of Procedures/Evaluations - *continued*

on sputum samples collected from participants. This assay will not be conducted under research conditions, but rather is conducted as part of routine South African standard of care, by the National Health Laboratory Services (NHLS). Sputum samples collected from participants will be couriered by NHLS couriers to NHLS laboratories for testing.

6.2.2 Special Assays or Procedures

No special assays or procedures are required for this study.

6.2.3 Specimen Collection, Preparation, Handling and Shipping

6.2.3.1 Instructions for Specimen Preparation, Handling, and Storage

Sputum collection is a routine clinical procedure that does not place the participant at any risk, and will be conducted by study staff only at household visits—the remainder of sputum collection for the study will be conducted by local clinic staff according to the standard of care. The participant will be instructed to go outside to provide the sputum sample. They should take several deep breaths, and hold each for 5 seconds, then cough such that sputum, if any, comes into their mouth. The participant then spits the sputum into the sputum collection jar and seals the lid on top, before handing to the study staff who then labels the specimen with participant details according to South African Department of Health standard procedures and places it inside of a sealed plastic bag then into a cooler until it is handed off to the NHLS courier at the local clinic for transport to the NHLS laboratory. At the laboratory, the sputum is processed immediately and tested by the Xpert MTB/RIF assay. No specimen is retained by the laboratory after testing

6.2.3.2 Specimen Shipment

Sputum specimens in closed vials inside of a sealed bag are transported by NHLS courier to the closest NHLS laboratory according to standard NHLS procedures. This occurs 1-2 times on each weekday, and the timing is variable by clinic. Specimens are kept at 4°C in a cooler until reaching the laboratory.

Appendix A: Schedule of Procedures/Evaluations - *continued*

7 STUDY SCHEDULE

7.1 Screening and Enrollment

Participants are identified either through attendance at a clinic (facility-based screening arm), through the TB treatment registers at clinics (contact tracing arm), through self-referral (incentive based contacts) or through presence at the household of a newly diagnosed TB case (household tracing arm). Participants identified in this way will be screened for eligibility at the time of obtaining consent.

This study encompasses a mixture of types of consent for different study components.

Informed consent (or for children>7 years, assent) forms (written) are attached for the following study components: in depth interviews, focus group discussions, interviews with index cases in contact referral arm, interviews with contacts in contact referral arm. Briefly, study staff ensure that the consent process takes place privately. Staff read from the written consent form to the participant, stopping frequently to ask if there are any questions. At the end, the staff allows time for additional questions and to ensure that the participant has understood the consent, before having the potential participant decline or accept participation.
Verbal consent will be used to obtain consent telephonically from participants in the facility-based screening arm who are interviewed by study staff, as well as to initially obtain consent for index cases in the contact referral arm (in order to establish meeting time to obtain written consent and give referral coupons in the incentive-based contact referral arm, and to schedule a household visit during which written consent can be obtained in the household tracing arm). Verbal consent is sought in these cases as the clinics in this rural district are spread over a large geographic area and have low patient volumes making it impractical to station a study staff member at each of the 56 clinics during the intervention periods. The verbal consent script is identical to the written consent script, and the consent process occurs in identical fashion, but over the phone. Verbal consent will be documented by the staff member obtaining that consent.

A waiver of consent has been granted by the Human Research Ethics Committee at the University of the Witwatersrand for the following groups: the three prospective file reviews (pre-intervention period, washout and post-intervention period and review of 90% of those testing Xpert negative in the facility-based screening arm), and those being screened for TB in the

Appendix A: Schedule of Procedures/Evaluations - *continued*

facility-based screening arm. We justify the waiver of informed consent for these individuals for the following reasons:

1. The research involves no more than minimal risk to subjects;

2. The waiver or alteration will not adversely affect the rights and welfare of the subjects;

3. The research could not practicably be carried out without the waiver or alteration. It is not feasible from a staffing and budgetary standpoint, as it would require a fulltime dedicated staff member at each of the 26 facility-based screening arm clinics; and

4. Whenever appropriate, the subjects will be provided with additional pertinent information after participation. We plan to disseminate research findings to participants through posters displayed at each study clinic.

5. For participants in the file review, we will be reviewing routinely collected data as part of the clinic's standard of care follow-up of patients who are symptomatic for TB. We do not collect any patient identifiers, therefore provision of written consent, including the participant's name represents an increase in risk to the participant. It is impractical to obtain consent from each clinic attendee who is investigated or diagnosed with TB at 56 clinics over the 1-2 year study period.

For those being screened for TB in the facility-based screening arm, the intervention involves performing a verbal TB symptom screen on individuals seeking health care. This intervention is low risk, and no data will be collected during this intervention, therefore provision of written consent, including the participant's name represents an increase in risk. It is also not feasible to collect written informed consent from all clinic attendees at 28 clinics over an 18 month period for the symptom screen. Those who are symptomatic and can provide sputum will be informed that they may be approached later for study participation

7.2 Enrollment/Baseline, if applicable

Not applicable.

7.3 Follow-up and Final Visits, if applicable

Not applicable.

Appendix A: Schedule of Procedures/Evaluations - *continued*

7.4 Early Termination Visit, if applicable

Not applicable.

7.5 Criteria for Discontinuation or Withdrawal of a Subject (or a Cohort), if applicable

Participants may withdraw from the study voluntarily at any time, but there are no further criteria for discontinuation or withdrawal.

Appendix A: Schedule of Procedures/Evaluations - *continued*

8 ASSESSMENT OF OUTCOME MEASURES

8.1 Specification of the Appropriate Outcome Measures

8.1.1 Primary Outcome Measures

Primary Objective: To measure the comparative effectiveness of active TB case finding in a rural high-burden setting

Our primary outcome measure for the analysis will be the number of TB cases in each cluster started on TB treatment. If necessary (i.e., if constrained randomization does not result in balanced groups in terms of pre-intervention patient volume), this number will be adjusted for the number of TB cases started on treatment in each cluster before the intervention was implemented.

8.1.2 Secondary Outcome Measures

Secondary Objective 1: To evaluate the acceptability and adoption of active TB case finding strategies in rural South Africa

Our primary outcome measure for this objective will come from a series of in-depth interviews (IDIs) with key informants, including TB health care workers and patients and community members who comprise the target populations of interest, including: (1) index TB patients who would be given vouchers; (2) household members of TB patients; (3) community members of high-TB-incidence neighborhoods;(4) individuals presenting to study clinics for routine care; and (5) health care workers involved in TB service provision. We plan to conduct up to 5 IDIs per each of these 5 subgroups for a total of 25 IDIs. Each semi-structured, in-depth interview will be approximately 45 minutes in length and will cover the following domains: individuals experiences with TB symptoms prior to diagnosis, the TB diagnostic process, clinic experiences and quality of TB care, feasibility and acceptability of different methods of ACF in early case detection, perceived benefits (e.g., reduced symptom burden, reduced transmission, confidentiality) and potential unintended consequences (e.g., stigma, inconvenience, overdiagnosis) of active TB case finding methods.

Secondary Objective 2: To describe the comparative implementation of facility-based screening and contact investigation

Appendix A: Schedule of Procedures/Evaluations - *continued*

We will measure the outcome for this objective using the RE-AIM (Reach, Effectiveness, Adoption, Implementation, and Maintenance) framework.[61] We will measure *reach* as the ab/solute number, proportion, and representativeness of individuals who accept TB screening under each ACF strategy. In the household screening arm, we will specifically also compare characteristics of index cases between those whose households do and do not agree to participate in screening. We will measure *effectiveness* as the yield of each strategy, described in the primary objective. We will measure *adoption* as the absolute number, proportion, and representativeness of study clinics which achieve high reach (defined as reaching >75% of eligible participants). We will assess clinic-level factors including size, number of TB patients, number and training of staff, and surrounding population density to evaluate predictors of high reach, though our power for this assessment will be low for all but the largest quantitative associations. We will measure *implementation* as fidelity to each ACF protocol, specifically using the indicators of: (a) completion of screening within one month of contact [either index case presentation or visit to healthcare facility]; and (b) initiation of treatment for individuals testing positive for TB, within 2 weeks of screening. For contact investigation, we will assess implementation through retrospective review of TB registers to assess the completeness of screening. For facility-based screening, we will assess implementation through retrospective review of medical charts (for comparability) and through direct observation in clinics on selected days (for increased fidelity to the target measurement).

<u>Secondary Objective 3: Evaluate the comparative costs and cost-effectiveness of each case-finding strategy</u>

The primary outcome for this analysis will be the incremental cost-effectiveness ratio, defined as (cost of ACF strategy 2 – cost of ACF strategy 1 [or no ACF])/(effectiveness of ACF strategy 2 – effectiveness of ACF strategy 1 [or no ACF]), where effectiveness is modeled as the number of disability-adjusted life years (DALYs) averted by the intervention.

Appendix A: Schedule of Procedures/Evaluations - *continued*

9 SAFETY ASSESSMENT AND REPORTING

9.1 Definition of Adverse Event (AE)

As our intervention is the implementation and observation of routine South African standard of care (intensified screening for TB at clinics and contact tracing of newly identified TB cases), we do not anticipate any medical adverse events which may occur as a result of this research study. Therefore, AEs (including SAEs) will not be recorded and reported for this study.

9.2 Definition of Serious Adverse Event (SAE)

N/A—see 9.1 “Definition of Adverse Event”

9.3 Reporting Procedures

N/A—see 9.1 “Definition of Adverse Event”

9.3.1 Serious Adverse Event Detection and Reporting

N/A—see 9.1 “Definition of Adverse Event”

9.3.2 Reporting of Pregnancy

This is a cross-sectional study which makes no attempt to exclude pregnant women. Pregnancy does not represent a complication within the context of this research study. Pregnancy status of any enrolled participant will be documented.

9.3.3 Procedures to be Followed in the Event of Abnormal Laboratory Test Values or Abnormal Clinical Findings

TB cases detected through this study are referred for care to their closest public health clinic for further evaluation and treatment, according to the South African standard of care. As TB is a measured outcome of this study, and detection of TB is the overall goal, it is not considered an AE or SAE.

Appendix A: Schedule of Procedures/Evaluations - *continued*

9.3.4 Type and Duration of the Follow-up of Subjects After Adverse Events

N/A—see 9.1 “Definition of Adverse Event”

9.4 Safety Oversight

Safety oversight will be conducted by a DSMB that is an independent group of experts that monitors subject safety and advises DMID. The DSMB members will be separate and independent of study personnel participating in this study and should not have scientific, financial or other conflict of interest related to the study. The DSMB will consist of members with appropriate expertise to contribute to the interpretation of the data from this study.

The DSMB will review study progress, including periodic assessments of data quality and timeliness, participant recruitment, accrual and retention. At specified times during the course of study as defined in the DSMB Charter.

- At least annually.
- Ad hoc when immediate concerns regarding observations during the study, or as needed.

The DSMB will operate under the rules of a DMID-approved charter that will be written at the organizational meeting of the DSMB. At this time, each data element that the DSMB needs to assess will be clearly defined. Procedures for DSMB reviews/meetings will be defined in the charter. The DSMB will review applicable data including the primary outcome (number of TB cases in each cluster started on TB treatment). Additional data may be requested by the DSMB, and interim statistical reports may be generated as deemed necessary and appropriate by DMID. The DSMB may receive data in aggregate and presented by group. The DSMB will meet and review this data at scheduled time points or ad hoc as needed during the study as defined in the DSMB charter. As an outcome of each review/meeting, the DSMB will make a recommendation as to the advisability of proceeding with study, and to continue, modify, or terminate the study.

Appendix A: Schedule of Procedures/Evaluations - *continued*

DMID or the DSMB chair may convene the DSMB on an ad hoc basis according to protocol criteria or if there are immediate concerns regarding observations during the course of the study.

Appendix A: Schedule of Procedures/Evaluations - *continued*

10 CLINICAL MONITORING STRUCTURE

Site monitoring is conducted to ensure that the human subject protections, study and laboratory procedures, study intervention administration, and data collection processes are of high quality and meet sponsor, ICH/GCP guidelines and applicable regulations, and that the study is conducted in accordance with the protocol, protocol-specific MOP and applicable sponsor standard operating procedures. DMID, the sponsoring agency, or its designee may conduct site-monitoring visits as detailed in the clinical monitoring plan.

Site visits may be made at standard intervals as defined by DMID and may be made more frequently as directed by DMID. Monitoring visits will include, but are not limited to, review of regulatory files, informed consent forms, laboratory reports, and protocol and GCP compliance. Site monitors will have access to the study site, study personnel, and all study documentation according to the DMID-approved site monitoring plan. Study monitors will meet with site principal investigators to discuss any problems and actions to be taken and document visit findings and discussions.

Appendix A: Schedule of Procedures/Evaluations - *continued*

11 STATISTICAL CONSIDERATIONS

11.1 Study Outcome Measures

Primary Objective: To measure the comparative effectiveness of active TB case finding in a rural high-burden setting

Our primary outcome measure for the analysis will be the number of TB cases in each cluster started on TB treatment. If necessary (i.e., if constrained randomization does not result in balanced groups in terms of pre-intervention patient volume), this number will be adjusted for the number of TB cases started on treatment in each cluster before the intervention was implemented. We will measure this outcome through record review of TB treatment registers at each study clinic.

Secondary Objective 1: To evaluate the acceptability and adoption of active TB case finding strategies in rural South Africa

Our primary outcome measure for this objective will come from a series of in-depth interviews (IDIs) with key informants, including TB health care workers and patients and community members who comprise the target populations of interest, including: (1) index TB patients who would be given vouchers; (2) household members of TB patients; (3) community members of high-TB-incidence neighborhoods;(4) individuals presenting to study clinics for routine care; and (5) health care workers involved in TB service provision. We plan to conduct up to 5 IDIs per each of these 5 subgroups for a total of 25 IDIs. Each semi-structured, in-depth interview will be approximately 45 minutes in length and will cover the following domains: individuals experiences with TB symptoms prior to diagnosis, the TB diagnostic process, clinic experiences and quality of TB care, feasibility and acceptability of different methods of ACF in early case detection, perceived benefits (e.g., reduced symptom burden, reduced transmission, confidentiality) and potential unintended consequences (e.g., stigma, inconvenience, overdiagnosis) of active TB case finding methods.

Secondary Objective 2: To describe the comparative implementation of facility-based screening and contact investigation

We will measure the outcome for this objective using the RE-AIM (Reach, Effectiveness, Adoption, Implementation, and Maintenance) framework.[61] We will measure *reach* as the absolute number, proportion, and representativeness of individuals who accept TB screening under each ACF strategy. In the household screening arm, we will specifically also compare characteristics of index cases between those whose households do and do not agree to participate in screening. We will measure *effectiveness* as the yield of each strategy, described in the primary objective. We will measure *adoption* as the absolute number, proportion, and representativeness of study clinics which achieve high reach

Appendix A: Schedule of Procedures/Evaluations - *continued*

(defined as reaching >75% of eligible participants). We will assess clinic-level factors including size, number of TB patients, number and training of staff, and surrounding population density to evaluate predictors of high reach, though our power for this assessment will be low for all but the largest quantitative associations. We will measure *implementation* as fidelity to each ACF protocol, specifically using the indicators of: (a) completion of screening within one month of contact [either index case presentation or visit to healthcare facility]; and (b) initiation of treatment for individuals testing positive for TB, within 2 weeks of screening. For contact investigation, we will assess implementation through retrospective review of TB registers to assess the completeness of screening. For facility-based screening, we will assess implementation through retrospective review of medical charts (for comparability) and through direct observation in clinics on selected days (for increased fidelity to the target measurement).

Secondary Objective 3: Evaluate the comparative costs and cost-effectiveness of each case-finding strategy
The primary outcome for this analysis will be the incremental cost-effectiveness ratio, defined as (cost of ACF strategy 2 – cost of ACF strategy 1 [or no ACF])/(effectiveness of ACF strategy 2 – effectiveness of ACF strategy 1), where effectiveness is modeled as the number of disability-adjusted life years (DALYs) averted by the intervention. The primary cost-effectiveness measure will be the incremental cost per DALY averted under facility-based versus contact-based screening, and the key secondary cost-effectiveness analysis will consider comparison of household-based versus incentive based strategies among the contact based screening arm. Costs will be measured prospectively from study data, and will comprehensively include those related to both health systems and patients (e.g. a societal perspective). Health systems costs will be captured using an "ingredients" approach as recommended by WHO-CHOICE,[64] through a combination of budgetary review, key informant interviews (e.g., administrative staff), direct observation (e.g., time-motion studies of patients and study staff), and prospective logbooks kept by study staff and other relevant personnel (e.g., clinic nurses) Patient-level costs will be measured through patient interview, using the Stop TB Partnership's "Tool to Estimate Patients' Costs" as a guide.[65] Costs from this approach will be compared (as a check) to a "top-down" approach in which we estimate large cost items (e.g., annual staff salaries) and divide those costs by the number of patients diagnosed and treated for TB under each strategy. Outcome measures will be derived by combining study data on patient characteristics (e.g., demographics, HIV status) and intervention effectiveness with literature estimates on key parameter values (e.g., disability weights[66], sensitivity of Xpert MTB/RIF[67], and notified data on treatment outcomes[1]) to construct a Markov model of a population of individuals who are eligible for TB screening under each strategy. In performing this evaluation, we will follow standard guidance for economic evaluation, including inflation using South Africa's GNI deflator, conversion to a common currency and year (2015 U.S. dollars), discounting of all future costs and effectiveness, and measurement of costs as economic (opportunity) costs.

Appendix A: Schedule of Procedures/Evaluations - *continued*

11.2 Sample Size Considerations

We base our sample size calculation on our primary comparison, namely contact investigation versus facility-based screening in a parallel design. We use calculations for cluster-randomized comparisons,[63] assuming a geometric mean "effective number of people at risk for TB" (i.e., individuals who would be evaluated for TB, were full screening procedures in place) of 1500 per clinic over a 1.5-year period. This number is only slightly higher than the number of Xpert MTB/RIF tests currently being performed in these clinics. Based on the notified prevalence of TB in Vhembe (300 per 100,000), we assume that, in the less-effective arm, 3% of these individuals would be found to have TB, for a yield of 45 cases per clinic over 18 months. We then consider the minimum important difference between ACF strategies to be a 25% increase in yield, which corresponds to a yield of 3.75% or 56 cases per clinic in the more-effective arm. Assuming a coefficient of variation (k) of 0.25 across clinics, we require 28 clusters per arm to detect this difference with 80% power. Importantly, we will have higher power for many of our secondary outcomes.

11.3 Participant Enrollment and Follow-Up

<u>By study arm, we expect our target sample size to break down as follows:</u>
In-depth interviews: 25 (accrued over a period of 4 months)
Focus group discussions: 30 (accrued over a period of 4 months)
Pre-intervention file review: 56,000 (accrued over a period of 1 year)
Index cases in contact-tracing arm: 3,150 (accrued over a period of 3 years)
Facility-based participants: 3,516 (accrued over a period of 18 months)
Washout period file review: 14,000 (accrued over a period of 6 months)
Post-intervention file review: 42,000 (accrued over a period of 2.5 years)
Household and close contacts in contact-tracing arm: 9,450 (accrued over a period of 3 years)

11.4 Analysis Plan

<u>Primary Objective: To measure the comparative effectiveness of active TB case finding in a rural high-burden setting</u>
Our primary analysis will utilize Poisson regression to model the outcome of the number of newly identified TB cases started on treatment. This model will account for within-cluster correlation while adjusting for both cluster-level covariates (including the number of TB notifications in each cluster prior to starting the intervention, HIV prevalence, and urban/rural setting) and individual-level covariates including age, sex, and prior TB history. We will compare this to a completely unadjusted analysis, reflecting the randomized nature of the study. The primary measure of association will be the relative yield of ACF (number of cases detected),

Appendix A: Schedule of Procedures/Evaluations - *continued*

comparing contact investigation to facility-based screening. In the analysis of the crossover component of this study, we will perform a cluster-level linear regression (weighted by the number of individuals within both periods) of the crossover differences in the yield of new TB cases starting TB treatment in the incentive-based versus household contact tracing arms.[62]

We will use simple descriptive epidemiologic analysis to describe and compare the individual patient characteristics of individuals diagnosed with TB under each ACF strategy. This will allow us to understand important differences in the target population (undiagnosed TB cases) reached by each strategy. Patients will be compared on relevant demographic and clinical characteristics such as age, gender, socio-economic status, previous TB history, length of TB symptoms, HIV status, antiretroviral therapy use, CD4 count, relationship and time spent with index case (for contact-based strategies) among others. We will perform these comparisons among facility-based versus contact-based strategies, as well as between incentive-based versus household based contact tracing. Comparisons will be made using differences in proportions and chi-square test for categorical characteristics, and t-tests for continuous characteristics of interest.

<u>Secondary Objective 1: To evaluate the acceptability and adoption of active TB case finding strategies in rural South Africa</u>
All IDIs will be audio-recorded, transcribed, translated as needed, and coded using the qualitative software Atlas.ti©. We will utilize content analysis using the constant comparative method to approach the interview transcripts and document salient themes that emerge from the IDIs, as well as participant narratives.[60] Based on initial findings from the IDIs, we will hold a series of focus group discussions (FGDs) among patients undergoing treatment for TB in the study clinics during the pre-intervention period. FGDs will examine group level perceptions of approaches to TB testing in the community, reasons why active TB case finding would or would not be perceived as feasible and acceptable, sources of TB-related stigma, perceptions of TB risk, and willingness to participate in the specific intervention strategies being studied here. We will conduct 3 FGDs with 8-10 individuals per group. Whereas the IDIs will focus on individual TB-related views and experiences, FGDs will examine group-level shared perceptions and norms surrounding TB and its detection and treatment, as well as specific areas of consensus or divergence regarding how to best operationalize each of the 3 ACF intervention strategies. Both in-depth interviews and focus group discussions will be conducted by trained qualitative research interviewers, using field guides established in consultation with the full study team.

<u>Secondary Objective 2: To describe the comparative implementation of facility-based screening and contact investigation</u>

As above in our primary objective, we will assess predictors of implementation through multivariable regression techniques (e.g., random-effects logistic regression to account for clustering at the clinic level) including demographic data, geographic (address) data, socioeconomic data, clinic-level data, and data on type and duration of symptoms as predictors. Costs and cost-effectiveness will be evaluated in Aim 4. Finally, we will measure maintenance as the extent to which ACF activities are integrated into routine practice, using as the primary

Appendix A: Schedule of Procedures/Evaluations - *continued*

indicator the proportional reach (i.e., proportion of eligible patients who undergo screening) of each intervention over the study period.

Secondary Objective 3: Evaluate the comparative costs and cost-effectiveness of each case-finding strategy

We will conduct one-way sensitivity analyses of all model parameters and multi-way sensitivity analyses of those parameters found to be most influential, in order to provide insight into the major drivers of cost-effectiveness and the settings in which each ACF intervention is likely to be preferred. We will also perform a probabilistic sensitivity analysis (using beta distributions for parameters with defined upper and lower bounds and gamma distributions for parameters defined from zero to infinity) to generate 95% uncertainty ranges around all estimates. We will construct cost-effectiveness acceptability curves, using as a reference South Africa's per-capita gross national income as a willingness to pay per DALY averted.[69]

11.5 Unblinding

In order not to delay publication of the results from the primary analysis, the US-based PI (David Dowdy), the South African-based PI (Neil Martinson) and NIH sponsors will be unblinded in a two-stage process as follows:

1. Following the completion of the first phase of the study (the first 18 months of enrollment into the facility-based screening and contact tracing arms) and cleaning and resolution of data queries, the study biostatistician will complete analysis of the primary study outcome measure (comparative yield [number of incident TB cases diagnosed and started on treatment] of facility-based screening versus contact investigation). Once this analysis has been finalized, results of this analysis alone will be unblinded to the PIs and NIH sponsors, for purposes of publishing the primary analysis in a timely fashion. A dataset containing sufficient data to run this primary analysis will be shared with the NIH at this time, if required. At this time, all results from the contact investigation arm will be reported without differentiation into household or incentive-based contact investigation, as data collection for that secondary analysis (using a crossover design) will remain ongoing at that time. Thus, the PIs and NIH sponsors will remain blinded to the identity and results of the household contact investigation and incentive-based contact investigation arms, as well as to the randomized assignment of intervention to study clinics.

2. Following the completing of the second phase of the study (the second 18 months of enrollment into the two contact tracing arms) and cleaning and resolution of data queries, the study biostatistician will complete the analysis of the secondary study outcome measure, the comparative yield of incentive-based versus household contact tracing. Once this analysis has been finalized, all results and intervention assignments will be unblinded.

Appendix A: Schedule of Procedures/Evaluations - *continued*

Appendix A: Schedule of Procedures/Evaluations - *continued*

12 ACCESS TO SOURCE DATA/DOCUMENTS

Research records for this study will be kept in a centralized study office, in compliance with Section 4.9 of ICH E6 GCP, and regulatory and institutional requirements for the protection of confidentiality of subjects. As part of participating in a DMID-sponsored, DMID-affiliated or manufacturer-sponsored study, each site will permit authorized representatives of the sponsor(s), DMID, and regulatory agencies to examine (and when required by applicable law, to copy) clinical records for the purposes of quality assurance reviews, audits and evaluation of the study safety and progress.

Appendix A: Schedule of Procedures/Evaluations - *continued*

13 QUALITY CONTROL AND QUALITY ASSURANCE

Details on all quality control and quality assurance procedures can be found in the associated DMID approved Clinical Quality Management Plan. The participating site is responsible for conducting routine quality assurance (QA) and quality control (QC) activities to internally monitor study progress and protocol compliance. The site principal investigator will provide direct access to all study-related sites, source data/data collection forms, and reports for the purpose of monitoring and auditing by the sponsor, and inspection by local and regulatory authorities. The site principal investigator will ensure all study personnel are appropriately trained and applicable documentations are maintained on site.

The data management center will implement quality control procedures beginning with the data entry system and generate data quality control checks that will be run on the database within 2 days of mobile data capture. Cyclical queries will be communicated to the site for clarification and resolution.

Protocol compliance will be managed on a day-to-day basis by the study coordinator, and overseen by the PI and other study investigators. The study coordinator will ensure proper training on study protocol of all study staff and will conduct routine field visits in order to monitor study compliance. Data quality will be monitored on a daily basis by the study coordinator who will review case report forms and remediate errors or omissions with study staff.

A study monitor from our local partner, the Perinatal HIV Research Unit, in South Africa will make periodic field visits to review study practices and documents in order to ensure compliance with ethical standards.

Appendix A: Schedule of Procedures/Evaluations - *continued*

14 ETHICS/PROTECTION OF HUMAN SUBJECTS

14.1 Declaration of Helsinki

The investigator will ensure that this study is conducted in full conformity with the current revision of the Declaration of Helsinki, or with the International Conference for Harmonisation Good Clinical Practice (ICH-GCP) regulations and guidelines, whichever affords the greater protection to the subject.

14.2 Institutional Review Board

Independent ethics review of this study will be handled in South Africa by the Human Research Ethics Committee at the University of the Witwatersrand, which holds a current U.S. Federal-Wide Assurance issued by OHRP. The institutional review board at Johns Hopkins Bloomberg School of Public Health has agreed to rely on the Human Research Ethics Committee at the University of the Witwatersrand as the institutional review board of record.

14.3 Informed Consent Process

Written consent (and assent)

Written consent will be obtained for all adults participating in focus group discussions and in depth interviews, which will only be performed with individuals over the age of 18. We will also obtain written consent (of both index cases and contacts) during household visits and (of index cases) during visits for delivery of vouchers in the contact tracing arm. Consent will be conducted privately. The study will be thoroughly explained to the participant in a language they can understand (English, Venda, Pedi or Tsonga). The participant will have a chance to ask questions about the study and will make an informed choice to participate or not participate in the study. Staff will emphasize that non-participation will not jeopardize any routine clinical care for the individual or their family. For subjects who speak and understand the language used in the consent document, but are unable to read or write, all of the information in the consent form will be communicated verbally, in the presence of an adult witness who is not a member of the study team; informed consent requires the signature or mark of the subject. If the eligible subject accepts to participate, he or she will sign or mark the informed consent document on the signature line of the consent form prior to any procedures being done specifically for the study. The witness will also sign and date the form, if the witness is confident that the subject has

Appendix A: Schedule of Procedures/Evaluations - *continued*

understood the explanation and is participating willingly. In addition, the witness will complete the date line for the subject. The subjects will have the opportunity to discuss the study with their surrogates or think about it prior to agreeing to participate. The subjects may withdraw consent at any time throughout the course of the trial. A copy of the signed and dated informed consent document will be given to the subjects for their records. The rights and welfare of the subjects will be protected by emphasizing, to subjects, that the quality of their medical care will not be adversely affected if they decline to participate in this study.

Verbal consent (and assent)
Verbal consent will be obtained for all adults participating in either facility-based screening who are interviewed (i.e., those participants who test positive for TB, as well as a representative sample of those who test negative)., Verbal consent will also be obtained for those participants enrolled as newly identified TB index cases and household or close contacts. The study will be explained verbally to the patient privately (either in a private room if study staff are present on-site, or on a private phone line, with a witness), if study staff are not on-site at the time of patient presentation) in a language they can understand (English, Venda, Pedi or Tsonga), and the participant will have a chance to ask questions and will make an informed choice to participate or not participate in the study. Potential participants will first be asked if they are interested to participate and then screened for eligibility. Study staff will inform the participant that their decision to participate or not is confidential and voluntary, and will not affect their clinical care in any way. Verbal consent will be documented by the staff member obtaining that consent.
For children, verbal consent will first be obtained from the parent/guardian, and then assent will be obtained from the child if 7 years or older. Verbal consent is being sought rather than written consent because the scientific benefit will be greater if we can enroll a representative sample of all patients (not just those who can return to clinic to provide written consent). The research also involves no more than minimal risk, and could not practicably be carried out with written consent, given the geographic dispersion of the clinics in the study. We currently have ethics permission from the IRB of record (the University of the Witwatersrand Human Research Ethics Committee) for verbal consent.

Waiver of consent
For individuals included in the pre-intervention, washout period or post-intervention file reviews, we will waive consent under the provision of 45 CFR 46.116(d), as this situation meets the following criteria:
1. The research involves no more than minimal risk to subjects;
2. The waiver or alteration will not adversely affect the rights and welfare of the subjects;
3. The research could not practicably be carried out without the waiver or alteration; and
4. Whenever appropriate, the subjects will be provided with additional pertinent information after participation.

Appendix A: Schedule of Procedures/Evaluations - *continued*

For these individuals, again, the scientific benefit increases if we are able to include all potential participants (not only those whom we could consent with on-site staff), and this activity of screening for TB is already the existing standard of care.

14.3.1 Informed Consent/Assent Process (in Case of a Minor or others unable to consent for themselves)

In the case of minors, aged 7-18 years, we will seek written or verbal assent, using a separate form from that used for adults. The assent forms will be written in simpler language the details of the study, study procedures and risks. In addition, we will seek parent/guardian informed consent for the participation of these minors. For children <7 years, we will seek only parental informed consent.

14.4 Exclusion of Women, Minorities, and Children (Special Populations)

This study does not intend to exclude any of these special populations.

14.5 Subject Confidentiality

The risks faced by participants in this study relate to consequences due to breach of confidentiality. Disclosure of any clinical information, including HIV and TB status, could be potentially damaging to the study participants. Household visits by the study team may engender stigma by neighbors. These risks will be minimized by ensuring that all study team members are trained in Good Clinical Practices and re-emphasizing the principles surrounding confidentiality on a regular basis in order to ensure that clinical information is handled with respect and in line with the highest research ethics standards. Focus groups will be conducted anonymously (no names will be collected or used and no identifying information will be collected). Participants who take part in focus groups will be advised to keep the discussion confidential but will be explicitly told in the informed consent process that there is no guarantee of confidentiality. One on one interviews will take place in a private room. No personally identifying information is collected in either focus groups or in one on one formative interviews. Recruitment, consent and interview of index cases will be conducted in one-on-one in a private room. Household visits will be conducted using unmarked cars and non-uniformed staff. For safety purposes, household visits will be conducted in teams of at least 2 study staff, who will present identifying badges and ask permission before entering the household. Patients in the facility-based arms will screened in private consultation rooms.

Appendix A: Schedule of Procedures/Evaluations - *continued*

All study materials will be kept in locked cabinets when not in use, and the study database and files containing transcribed focus groups and interviews will be password protected and stored on secure servers. All paper-based study data will be stored in locked cabinets in a locked room. Identifiers will be kept separately from all other study data, and will be coded using a unique study ID. The key for this study ID will likely be kept separately. The database will not contain participant identifiers, and will be password protected and stored on a secure database. Data will be accessed by JHU investigators through the secure online password protected database.

14.6 Future Use of Stored Specimens

N/A

Appendix A: Schedule of Procedures/Evaluations - *continued*

15 DATA HANDLING AND RECORD KEEPING

15.1 Data Management Responsibilities

Details of study data management can be found in the corresponding Data Management plan. In brief,cata collection will be conducted by research assistants, interviewers and study nurses. The study coordinator and data manager will be responsible for routine quality control of the data. The principal investigator and other study investigators will be responsible for periodic data review, generating study materials and reports. Our local partners, the Perinatal HIV Research Unit will be responsible for retaining source documents and records. The PI and investigators will be responsible for data interpretation, analysis, as well as review of tables and listing. Reporting to the sponsor and ethics/IRB committees will be the responsibility of the PI and other investigators.

15.2 Data Capture Methods

We anticipate using handheld mobile devices (phones and/or tablets) in order to collect data for this study. We will utilize REDCap, a secure mobile data collection platform for this purpose. Data from interviews and file reviews can be captured directly on the mobile device and either immediately uploaded to a secure online database, or uploaded later in case a good cellular signal is not available. Data may also be captured on paper forms then later entered into REDCap if mobile devices are not available/functional.

15.3 Types of Data

Types of data collected will include those from IDIs, FGD, chart and record review on above listed outcomes, as well as participant interviews.

15.4 Timing/Reports

Data review will occur on a quarterly basis in order to ensure enrollment targets are met, and that inclusion/exclusion criteria are followed. Final data review will take place at the completion of each study phase.

Appendix A: Schedule of Procedures/Evaluations - *continued*

15.5 Study Records Retention

The investigator will maintain records pertaining to this study for a period of 5 years following the close of enrollment. Permission is not required prior to destruction of records.

15.6 Protocol Deviations

We will monitor data on a quarterly basis in order to ensure that there are no deviations from protocol.

Protocol compliance will be managed on a day-to-day basis by the study coordinator, and overseen by the PI and other study investigators. The study coordinator will ensure proper training on study protocol of all study staff and will conduct routine field visits in order to monitor study compliance. Additionally, she will review consent forms and study case report forms to identify potential areas for protocol violation, and conduct retraining as necessary. The study PI and other investigators will make period study monitoring visits to ensure protocol adherence, and will conduct weekly teleconference calls with the study coordinator to review progress and address protocol issues in a timely fashion. Likewise, the study coordinator will monitor day-to-day compliance with ethical standards through training, review of practices, consents, enrollment logs and case report forms, as well as through periodic unannounced field visits. Additionally, a study monitor from our local partner, the Perinatal HIV Research Unit, in South Africa will make periodic field visits to review study practices and documents in order to ensure compliance with ethical standards.

Appendix A: Schedule of Procedures/Evaluations - *continued*

16 PUBLICATION POLICY

Following completion of the study, the investigator may publish the results of this research in a scientific journal. The International Committee of Medical Journal Editors (ICMJE) member journals have adopted a trials-registration policy as a condition for publication. This policy requires that all clinical trials be registered in a public trials registry. We will register this trial at the appropriate local registry, the South African National Clinical Trials Register (www.sanctr.gov.za). Other biomedical journals are considering adopting similar policies. Any clinical trial starting enrollment after 01 July 2005 must be registered either on or before the onset of patient enrollment. For trials that began enrollment prior to this date, the ICMJE member journals will require registration by 13 September 2005 before considering the results of the trial for publication.

The ICMJE defines a clinical trial as any research project that prospectively assigns human subjects to intervention or comparison groups to study the cause-and-effect relationship between a medical intervention and a health outcome. Studies designed for other purposes, such as to study pharmacokinetics or major toxicity (eg, Phase 1 trials), would be exempt from this policy.

Appendix A: Schedule of Procedures/Evaluations - *continued*

17 LITERATURE REFERENCES

1. WHO. Global Tuberculosis Report. Geneva, Switzerland; 2013.
2. WHO. Looking beyond 2015, Proposed Global Strategy and Targets for TB Prevention, Care and Control After 2015. 2014 [cited May 10, 2014]; Available from: http://www.who.int/tb/post2015_strategy/en/
3. Herbert N, George A, Baroness Masham of I, Sharma V, Oliver M, Oxley A, et al. World TB Day 2014: finding the missing 3 million. Lancet. 2014; 383(9922): 1016-8.
4. Dodd PJ, White RG, Corbett EL. Periodic active case finding for TB: when to look? PloS one. 2011; 6(12): e29130.
5. Murray CJ, Salomon JA. Modeling the impact of global tuberculosis control strategies. Proceedings of the National Academy of Sciences of the United States of America. 1998; 95(23): 13881-6.
6. Kasaie P, Andrews JR, Kelton WD, Dowdy DW. Timing of tuberculosis transmission and the impact of household contact tracing. An agent-based simulation model. American journal of respiratory and critical care medicine. 2014; 189(7): 845-52.
7. Khan AJ, Khowaja S, Khan FS, Qazi F, Lotia I, Habib A, et al. Engaging the private sector to increase tuberculosis case detection: an impact evaluation study. The Lancet infectious diseases. 2012; 12(8): 608-16.
8. Dowdy DW, Lotia I, Azman AS, Creswell J, Sahu S, Khan AJ. Population-level impact of active tuberculosis case finding in an Asian megacity. PloS one. 2013; 8(10): e77517.
9. Azman AS, Golub JE, Dowdy DW. How Much is a Case Worth? Estimating the Population-Level Impact and Cost-Effectiveness of Active Case Finding for Tuberculosis. Lancet Global Health. 2014; In revision.
10. Ayles H, Muyoyeta M, Du Toit E, Schaap A, Floyd S, Simwinga M, et al. Effect of household and community interventions on the burden of tuberculosis in southern Africa: the ZAMSTAR community-randomised trial. Lancet. 2013; 382(9899): 1183-94.
11. Kranzer K, Afnan-Holmes H, Tomlin K, Golub JE, Shapiro AE, Schaap A, et al. The benefits to communities and individuals of screening for active tuberculosis disease: a systematic review. The international journal of tuberculosis and lung disease : the official journal of the International Union against Tuberculosis and Lung Disease. 2013; 17(4): 432-46.
12. Golub JE, Mohan CI, Comstock GW, Chaisson RE. Active case finding of tuberculosis: historical perspective and future prospects. The international journal of tuberculosis and lung disease : the official journal of the International Union against Tuberculosis and Lung Disease. 2005; 9(11): 1183-203.
13. CDC. Guidelines for the investigation of contacts of persons with infectious tuberculosis. Recommendations from the National Tuberculosis Controllers Association and CDC. MMWR Recommendations and reports : Morbidity and mortality weekly report Recommendations and reports / Centers for Disease Control. 2005; 54(RR-15): 1-47.
14. Erkens CG, Kamphorst M, Abubakar I, Bothamley GH, Chemtob D, Haas W, et al. Tuberculosis contact investigation in low prevalence countries: a European consensus. The European respiratory journal. 2010; 36(4): 925-49.

Appendix A: Schedule of Procedures/Evaluations - *continued*

15. WHO. Recommendations for investigating contacts of persons with infectious tuberculosis in low- and middle-income countries. Geneva, Switzerland; 2012.
16. Shapiro AE, Variava E, Rakgokong MH, Moodley N, Luke B, Salimi S, et al. Community-based targeted case finding for tuberculosis and HIV in household contacts of patients with tuberculosis in South Africa. American journal of respiratory and critical care medicine. 2012; 185(10): 1110-6.
17. Corbett EL, Bandason T, Duong T, Dauya E, Makamure B, Churchyard GJ, et al. Comparison of two active case-finding strategies for community-based diagnosis of symptomatic smear-positive tuberculosis and control of infectious tuberculosis in Harare, Zimbabwe (DETECTB): a cluster-randomised trial. Lancet. 2010; 376(9748): 1244-53.
18. Hoffmann CJ, Variava E, Rakgokong M, Masonoke K, van der Watt M, Chaisson RE, et al. High prevalence of pulmonary tuberculosis but low sensitivity of symptom screening among HIV-infected pregnant women in South Africa. PloS one. 2013; 8(4): e62211.
19. O'Grady J, Bates M, Chilukutu L, Mzyece J, Cheelo B, Chilufya M, et al. Evaluation of the Xpert MTB/RIF assay at a tertiary care referral hospital in a setting where tuberculosis and HIV infection are highly endemic. Clinical infectious diseases : an official publication of the Infectious Diseases Society of America. 2012; 55(9): 1171-8.
20. Economics T. Rural population growth in sub Saharan Africa. 2014 [cited May 25, 2014]; Available from: http://www.tradingeconomics.com/sub-saharan-africa/rural-population-growth-annual-percent-wb-data.html
21. Dye C, Bourdin Trunz B, Lonnroth K, Roglic G, Williams BG. Nutrition, diabetes and tuberculosis in the epidemiological transition. PloS one. 2011; 6(6): e21161.
22. Hoa NB, Cobelens FG, Sy DN, Nhung NV, Borgdorff MW, Tiemersma EW. First national tuberculin survey in Viet Nam: characteristics and association with tuberculosis prevalence. The international journal of tuberculosis and lung disease : the official journal of the International Union against Tuberculosis and Lung Disease. 2013; 17(6): 738-44.
23. Tupasi TE, Radhakrishna S, Chua JA, Mangubat NV, Guilatco R, Galipot M, et al. Significant decline in the tuberculosis burden in the Philippines ten years after initiating DOTS. The international journal of tuberculosis and lung disease : the official journal of the International Union against Tuberculosis and Lung Disease. 2009; 13(10): 1224-30.
24. Zhao Y, Xu S, Wang L, Chin DP, Wang S, Jiang G, et al. National survey of drug-resistant tuberculosis in China. The New England journal of medicine. 2012; 366(23): 2161-70.
25. Andrews JR, Morrow C, Walensky RP, Wood R. Integrating Social Contact and Environmental Data in Evaluating Tuberculosis Transmission in a South African Township. The Journal of infectious diseases. 2014.
26. Verver S, Warren RM, Munch Z, Richardson M, van der Spuy GD, Borgdorff MW, et al. Proportion of tuberculosis transmission that takes place in households in a high-incidence area. Lancet. 2004; 363(9404): 212-4.
27. Salaniponi FM, Harries AD, Banda HT, Kang'ombe C, Mphasa N, Mwale A, et al. Care seeking behaviour and diagnostic processes in patients with smear-positive pulmonary tuberculosis in Malawi. The international journal of tuberculosis and lung disease : the official journal of the International Union against Tuberculosis and Lung Disease. 2000; 4(4): 327-32.
28. Becerra MC, Pachao-Torreblanca IF, Bayona J, Celi R, Shin SS, Kim JY, et al. Expanding tuberculosis case detection by screening household contacts. Public Health Rep. 2005; 120(3): 271-7.

Appendix A: Schedule of Procedures/Evaluations - *continued*

29. Dowdy DW, Chaisson RE. Post-2015 tuberculosis strategies in a pre-2015 world. The international journal of tuberculosis and lung disease : the official journal of the International Union against Tuberculosis and Lung Disease. 2013; 17(2): 143.
30. Dowdy DW, Lourenco MC, Cavalcante SC, Saraceni V, King B, Golub JE, et al. Impact and cost-effectiveness of culture for diagnosis of tuberculosis in HIV-infected Brazilian adults. PloS one. 2008; 3(12): e4057.
31. Golub JE, Dowdy DW. Screening for active tuberculosis: methodological challenges in implementation and evaluation. The international journal of tuberculosis and lung disease : the official journal of the International Union against Tuberculosis and Lung Disease. 2013; 17(7): 856-65.
32. Theron G, Zijenah L, Chanda D, Clowes P, Rachow A, Lesosky M, et al. Feasibility, accuracy, and clinical effect of point-of-care Xpert MTB/RIF testing for tuberculosis in primary-care settings in Africa: a multicentre, randomised, controlled trial. Lancet. 2014; 383(9915): 424-35.
33. Choi HW, Miele K, Dowdy D, Shah M. Cost-effectiveness of Xpert(R) MTB/RIF for diagnosing pulmonary tuberculosis in the United States. The international journal of tuberculosis and lung disease : the official journal of the International Union against Tuberculosis and Lung Disease. 2013; 17(10): 1328-35.
34. Dowdy DW, O'Brien MA, Bishai D. Cost-effectiveness of novel diagnostic tools for the diagnosis of tuberculosis. The international journal of tuberculosis and lung disease : the official journal of the International Union against Tuberculosis and Lung Disease. 2008; 12(9): 1021-9.
35. Millman AJ, Dowdy DW, Miller CR, Brownell R, Metcalfe JZ, Cattamanchi A, et al. Rapid molecular testing for TB to guide respiratory isolation in the U.S.: a cost-benefit analysis. PloS one. 2013; 8(11): e79669.
36. Owens JP, Fofana MO, Dowdy DW. Cost-effectiveness of novel first-line treatment regimens for tuberculosis. The international journal of tuberculosis and lung disease : the official journal of the International Union against Tuberculosis and Lung Disease. 2013; 17(5): 590-6.
37. Sun D, Dorman S, Shah M, Manabe YC, Moodley VM, Nicol MP, et al. Cost utility of lateral-flow urine lipoarabinomannan for tuberculosis diagnosis in HIV-infected African adults. The international journal of tuberculosis and lung disease : the official journal of the International Union against Tuberculosis and Lung Disease. 2013; 17(4): 552-8.
38. Dowdy DW, Basu S, Andrews JR. Is passive diagnosis enough? The impact of subclinical disease on diagnostic strategies for tuberculosis. American journal of respiratory and critical care medicine. 2013; 187(5): 543-51.
39. Dowdy DW, Chaisson RE, Moulton LH, Dorman SE. The potential impact of enhanced diagnostic techniques for tuberculosis driven by HIV: a mathematical model. AIDS. 2006; 20(5): 751-62.
40. Dowdy DW, Davis JL, den Boon S, Walter ND, Katamba A, Cattamanchi A. Population-level impact of same-day microscopy and Xpert MTB/RIF for tuberculosis diagnosis in Africa. PloS one. 2013; 8(8): e70485.
41. Dowdy DW, Golub JE, Chaisson RE, Saraceni V. Heterogeneity in tuberculosis transmission and the role of geographic hotspots in propagating epidemics. Proceedings of the National Academy of Sciences of the United States of America. 2012; 109(24): 9557-62.

Appendix A: Schedule of Procedures/Evaluations - *continued*

42. Dowdy DW, Golub JE, Saraceni V, Moulton LH, Cavalcante SC, Cohn S, et al. Impact of Isoniazid Preventive Therapy for HIV-Infected Adults in Rio de Janeiro, Brazil: An Epidemiological Model. J Acquir Immune Defic Syndr. 2014.
43. Sun AY, Pai M, Salje H, Satyanarayana S, Deo S, Dowdy DW. Modeling the impact of alternative strategies for rapid molecular diagnosis of tuberculosis in Southeast Asia. American journal of epidemiology. 2013; 178(12): 1740-9.
44. Deluca A, Chaisson RE, Martinson NA. Intensified case finding for tuberculosis in prevention of mother-to-child transmission programs: a simple and potentially vital addition for maternal and child health. J Acquir Immune Defic Syndr. 2009; 50(2): 196-9.
45. Golub JE, Pronyk P, Mohapi L, Thsabangu N, Moshabela M, Struthers H, et al. Isoniazid preventive therapy, HAART and tuberculosis risk in HIV-infected adults in South Africa: a prospective cohort. AIDS. 2009; 23(5): 631-6.
46. Gounder CR, Wada NI, Kensler C, Violari A, McIntyre J, Chaisson RE, et al. Active tuberculosis case-finding among pregnant women presenting to antenatal clinics in Soweto, South Africa. J Acquir Immune Defic Syndr. 2011; 57(4): e77-84.
47. Hanrahan CF, Golub JE, Mohapi L, Tshabangu N, Modisenyane T, Chaisson RE, et al. Body mass index and risk of tuberculosis and death. AIDS. 2010; 24(10): 1501-8.
48. Kasambira TS, Shah M, Adrian PV, Holshouser M, Madhi SA, Chaisson RE, et al. QuantiFERON-TB Gold In-Tube for the detection of Mycobacterium tuberculosis infection in children with household tuberculosis contact. The international journal of tuberculosis and lung disease : the official journal of the International Union against Tuberculosis and Lung Disease. 2011; 15(5): 628-34.
49. Martinson NA, Barnes GL, Moulton LH, Msandiwa R, Hausler H, Ram M, et al. New regimens to prevent tuberculosis in adults with HIV infection. The New England journal of medicine. 2011; 365(1): 11-20.
50. Martinson NA, Moultrie H, van Niekerk R, Barry G, Coovadia A, Cotton M, et al. HAART and risk of tuberculosis in HIV-infected South African children: a multi-site retrospective cohort. The international journal of tuberculosis and lung disease : the official journal of the International Union against Tuberculosis and Lung Disease. 2009; 13(7): 862-7.
51. Otwombe KN, Variava E, Holmes CB, Chaisson RE, Martinson N. Predictors of delay in the diagnosis and treatment of suspected tuberculosis in HIV co-infected patients in South Africa. The international journal of tuberculosis and lung disease : the official journal of the International Union against Tuberculosis and Lung Disease. 2013; 17(9): 1199-205.
52. Hanrahan CF, Dorman SE, Erasmus L, Koornhof H, Coetzee G, Golub JE. The impact of expanded testing for multidrug resistant tuberculosis using genotype [correction of geontype] MTBDRplus in South Africa: an observational cohort study. PloS one. 2012; 7(11): e49898.
53. Hanrahan CF, Selibas K, Deery CB, Dansey H, Clouse K, Bassett J, et al. Time to treatment and patient outcomes among TB suspects screened by a single point-of-care xpert MTB/RIF at a primary care clinic in Johannesburg, South Africa. PloS one. 2013; 8(6): e65421.
54. Hanrahan CF, Theron G, Bassett J, Dheda K, Scott L, Stevens W, et al. Xpert MTB/RIF as a measure of sputum bacillary burden: variation by HIV status and immunosuppression. American journal of respiratory and critical care medicine. 2014.
55. Lippincott CK, Miller MB, Popowitch EB, Hanrahan CF, Van Rie A. Xpert MTB/RIF Assay Shortens Airborne Isolation for Hospitalized Patients With Presumptive Tuberculosis in

Appendix A: Schedule of Procedures/Evaluations - *continued*

the United States. Clinical infectious diseases : an official publication of the Infectious Diseases Society of America. 2014.
56. Van Rie A, Hanrahan C. Active case finding for tuberculosis: what is the most informative measure for policy makers? The international journal of tuberculosis and lung disease : the official journal of the International Union against Tuberculosis and Lung Disease. 2014; 18(3): 377.
57. Omar T, Schwartz S, Hanrahan C, Modisenyane T, Tshabangu N, Golub JE, et al. Progression and regression of premalignant cervical lesions in HIV-infected women from Soweto: a prospective cohort. AIDS. 2011; 25(1): 87-94.
58. Dowdy DW, Chaisson RE, Maartens G, Corbett EL, Dorman SE. Impact of enhanced tuberculosis diagnosis in South Africa: a mathematical model of expanded culture and drug susceptibility testing. Proceedings of the National Academy of Sciences of the United States of America. 2008; 105(32): 11293-8.
59. South African DOH. Guidelines for the diagnosis of tuberculosis. Pretoria, South Africa; 2013.
60. Denzin N, Lincoln Y. Handbook of Qualitative Research. 2nd edition ed. Thousand Oaks, CA: Sage Publications; 2000.
61. Glasgow RE, Vogt TM, Boles SM. Evaluating the public health impact of health promotion interventions: the RE-AIM framework. American journal of public health. 1999; 89(9): 1322-7.
62. Turner RM, White IR, Croudace T. Analysis of cluster randomized cross-over trial data: a comparison of methods. Statistics in medicine. 2007; 26(2): 274-89.
63. Hayes RJ, Bennett S. Simple sample size calculation for cluster-randomized trials. International journal of epidemiology. 1999; 28(2): 319-26.
64. WHO guide to cost-effectiveness analysis. Geneva, Switzerland; 2003.
65. KNCV Tuberculosis Foundation. Tool to estimate patients' costs. Geneva, Switzerland; 2008.
66. Murray CJ, Vos T, Lozano R, Naghavi M, Flaxman AD, Michaud C, et al. Disability-adjusted life years (DALYs) for 291 diseases and injuries in 21 regions, 1990-2010: a systematic analysis for the Global Burden of Disease Study 2010. Lancet. 2012; 380(9859): 2197-223.
67. Steingart KR, Sohn H, Schiller I, Kloda LA, Boehme CC, Pai M, et al. Xpert(R) MTB/RIF assay for pulmonary tuberculosis and rifampicin resistance in adults. The Cochrane database of systematic reviews. 2013; 1: CD009593.
68. Drummond MF, Sculpher MJ, Torrance GW, O'Briend BJ, Stoddart GL. Methods for the economic evaluation of helath care programmes. 3rd edition ed. New York, NY: Oxford University Press; 2005.
69. The World Bank. GNI per capita, Atlas method (curent US$). 2014 [cited May 25, 2014]; Available from: http://data.worldbank.org/indicator/NY.GNP.PCAP.CD/countrie
