# Supplementary material for: Contact tracing versus facility-based screening for active TB case finding in rural South Africa: A pragmatic cluster-randomized trial (Kharitode TB)
Source: PLoS Med. 2019 Apr 30;16(4):e1002796. doi: 10.1371/journal.pmed.1002796 (PMC6490908; doi:10.1371/journal.pmed.1002796)
Supplement: S2 Text — This file contains the questionnaires administered to all participants: TB index patients and their close contacts. TB, tuberculosis. (PDF) [file pmed.1002796.s002.pdf]

Form 2: Facility Based Arm Interview

To be completed for every consenting participant who tests Xpert-positive, plus a consecutive sample of those testing negative, in the facility-based arm

**Question**	**Response Elements**
1. Name of facility	1-56 (depending on specific facilities included)
2. Date of interview	DD/MM/YYYY
3. Data collector ID	0-99
5. What is your age, in years?	0-99
6. Record the patient's sex.	1. Male<br>2. Female
7. Study ID	Number study ID
8. Is the participant a patient recently tested for TB with Xpert?	1. Yes<br>2. No
9. Has informed consent been provided by the participant if >18 years of age?	1. Yes<br>2. No
10. Has parental consent and adolescent/child assent been provided if the participant is <18 years of age?	1. Yes<br>2. No
11. Have you been interviewed for this study before?	1. Yes<br>2. No<br>77. Don't know
12. I want you to think back to the day when you originally came to clinic and were asked to give a specimen for a TB test (by coughing in a cup). What was the <u>primary</u> reason that you went to clinic that day?	1. I have a long-term condition such as HIV or cancer, and I was coming for regular care.<br>2. I had a cough, fever, sweats, or weight loss, and was coming for a diagnosis of those symptoms.<br>3. I was coming for diagnosis or treatment of other short-term symptoms.<br>4. I was coming for a regular check-up (or for antenatal care), not because I thought I was sick at all.<br>5. I was coming for medications, advice, or some other reason not listed above.<br>6. I was accompanying someone else.<br>9. No answer/don't know
13. On that day, which of the following symptoms did you have? *Select all that apply.*	1. Cough<br>2. Fever<br>3. Weight loss (more than 5 kg, or enough to make my clothes loose)<br>4. Drenching sweats at night<br>5. Pain in my chest<br>6. Pain in another part of my body<br>7. Skin problem (for example, rash)

	8. Stomach or intestinal problem (for example, nausea or diarrhea)<br>9. Genital or urinary problem (for example, pain on urination, genital lesion)<br>10. None of the above<br>11. No symptoms (for example, came for routine checkup or accompanying someone else)
14. Which of your symptoms did you notice first?<br>*Select only one.*	1. Cough<br>2. Fever<br>3. Weight loss (more than 5 kg, or enough to make my clothes loose)<br>4. Drenching sweats at night<br>5. Pain in my chest<br>6. Pain in another part of my body<br>7. Skin problem (for example, rash)<br>8. Stomach or intestinal problem (for example, nausea or diarrhea)<br>9. Genital or urinary problem (for example, pain on urination, genital lesion)<br>10. None of the above
15. How long had you had that symptom before you came to clinic that day?	0-10 years, 0-12 months, 0-4 weeks, 0-7 days<br>77 for unknown
16. If you had fever, cough, weight loss, or night sweats, but they were not the first symptom you noticed, how long did you have them?	0-10 years, 0-12 months, 0-4 weeks, 0-7 days<br>77 Unknown
17. Thinking about the time when you had these symptoms and were sick, to what extent did his illness affect your household **financially**?	1. No impact<br>2. Little impact<br>3. Moderate impact<br>4. Serious impact<br>5. Very serious impact
18. On that day, approximately how long did it take you to get to clinic?	0-24 hours, 0-60 minutes
19. What was your primary mode of transport to the clinic (meaning the type of transport that took you the longest distance)?	1. Walking<br>2. Bus<br>3. Taxi<br>4. Motorbike<br>5. Car<br>6. Tuk-tuk<br>7. Bicycle<br>9. Other
20. How much do you estimate that a one-way trip to the clinic cost you?	R 0 – 10,000

21. On that day, how much time did it take for you to get to the clinic, starting from when you left your house?	0-24 hours, 0-60 minutes
22. On that day, how much TOTAL time did you spend for your visit to clinic, from the time that you left your home until the time you returned home?	0-24 hours, 0-60 minutes
23. Please estimate the amount of money you and your household members spent on that visit to the clinic that you would not have had to spend if you never came to clinic. For example: transportation, childcare, any food you had to purchase that you would not have bought at home or work, and the cost of any medicines. Please include the value of any goods or services you have had to sell or exchange, and any money that you have had to borrow from someone else, as well as the value of any missed work. Please also include the costs for anyone else who might have accompanied you to clinic today.	R 0 – 10,000
24. Before that visit, how long had it been since you had last visited this clinic (or another clinic like this one), for any reason?	0-10 years, 0-12 months, 0-4 weeks, 0-7 days<br>66 never<br>77 Unknown
25. How many visits did you make to seek care or advice for TB symptoms (cough, fever, night sweats, or weight loss) including being admitted to the hospital? (Include informal providers such as herbalists, chemists, and formal providers such as emergency care, private clinics, government health centers)	0-6+ visits
26. Taking all of these visits together, plus any other costs to manage your TB symptoms (for example: cough medicine, special foods such as meats or energy drinks, vitamins, caregivers), please estimate the TOTAL amount of money you and your household members have had to spend to manage your symptoms since they started, that you would not have had to spend if you never got sick. Please include the value of any goods or services you have had to sell or exchange, and any money	R 0 – 10,000

that you have had to borrow from someone else.	
27. In addition to what you have spent above, please also estimate the TOTAL amount of money that you would have earned, that you did not earn because of your TB symptoms. Please include both lost time from work because you were sick, and time that you missed from working because you had to visit clinics or see other healthcare providers. As above, please also include the value of any goods or services you have had to sell or exchange, and any money that you have had to borrow from someone else.	R 0 – 10,000
28. In which village or town do you live?	(responses based on available towns)
29. What is the highest grade of education that you have attained?	0. None<br>1-12. Based on grades<br>13. Any postgraduate education<br>14. Attained postgraduate degree
30. Which of the following best describes your occupation?	1. Self-employed<br>2. Student<br>3. Salaried worker<br>4. Occasional work (piece jobs)<br>5. Unemployed but able to work<br>6. Unemployed and unable to work
31. Not including you, how many people in your household work?	0-20
32. Do you smoke tobacco?	1. Yes<br>2. Not currently, but formerly<br>3. No, never<br>88. Refused
33. For how many years have you smoked/did you smoke?	0-99 years<br>77 unknown
34. About how many cigarettes (or their equivalent) do you smoke, or did you smoke, each day?	0-999
35. Do you have any other medical conditions? Please check all that apply.	1. Obstructive lung disease (asthma or COPD)<br>2. Diabetes<br>3. Gastroesophageal reflux<br>4. Seasonal allergies<br>5. Silicosis or other destructive lung disease<br>6. Lung cancer

	7. Any other form of cancer<br>8. High blood pressure<br>9. High cholesterol<br>10. HIV or AIDS<br>11. Other<br>88. Refused
36. Have you ever been diagnosed with TB in the past?	1. Yes<br>2. No<br>77. Don't know
37. Approximately how long has it been since you were treated for TB in the past, if you ever were?	0-99 years
38. Do you know your HIV status, and if you are comfortable to will you please share it with me?	1. Positive<br>2. Negative<br>77. Unknown<br>88. Refused
39. How long ago was the last time you were tested for HIV?	0-10 years, 0-12 months, 0-4 weeks, 0-7 days<br>66 never<br>77 n/a or unknown
40. Are you currently taking antiretrovirals for your HIV?	1. Yes<br>2. No<br>77. Don't know or Not applicable
41. If you were to develop a mild cough, how long would it likely be before you saw a doctor or other healthcare professional for a diagnosis?	0-12 months, 0-4 weeks, 0-7 days<br>77 n/a or unknown
42. Once you did decide to see a doctor or other professional, where would you go?	1. Pharmacy<br>2. Traditional healer<br>3. This clinic<br>4. A different public clinic<br>5. Private clinic<br>6. Public hospital<br>7. Private hospital<br>8. Other
43. What is your feeling about getting tested for TB, even if you were coming to clinic for a completely different reason?	1. I am glad to be tested.<br>2. I am willing to be tested.<br>3. I don't care whether I am tested or not.<br>4. I prefer not to be tested.<br>5. I would refuse to be tested.
44. How many people, not including yourself, live in your household? Please include everyone- adults and children – who sleep here at least one night a week. Please do not include guests and temporary visitors.	0-99

45. Does anyone in your house have TB, or been told they have TB in the last 2 years?	1. Yes<br>2. No<br>77. Don't know
46. Who is the head of your household?	1. Myself<br>2. Male >60 years<br>3. Male 18-60 years<br>4. Male child <18 years<br>5. Female >60 years<br>6. Female 18-60 years<br>7. Female child <18 years
47. If you are not the head of the household, what is the level of education of the head of the household?	0. None<br>1-12. Based on grades<br>13. Any postgraduate education<br>14. Attained postgraduate degree
48. How long have you lived in your current household?	1. Less than 1 year<br>2. 1-2 years<br>3. 3-5 years<br>4. More than 5 years<br>77. Unknown
49. What is your household's estimated monthly income from each of the following sources:<br>a. Regular employment<br>b. Casual work<br>c. Grant/pensions<br>d. Other sources (traded goods, investments, etc.)	 <br>R 0 – 100,000<br>R 0 – 100,000<br>R 0 – 100,000<br>R 0 – 100,000<br><br>77. Unknown<br>88. Refused
50. Please think back to the time before you were sick. Can you estimate your monthly income at that time? If you were paid in-kind or received payment not in cash (for example, for crops), please estimate how much cash you would receive if you sold those goods on the market today.	R 0 – 100,000
51. If you had to sell all of your belongings and all of your household members' belongings (including your house, land, and any other valuables), how	R 0 – 1,000,000

much money would you be able to get for your entire household's belongings?	

Form 4: Household Contact Arm Contact Interview

To be completed for every consenting contact in the household contact tracing arm

**Question**	**Response Elements**
1. Name of facility	1-56 (depending on specific facilities included)
2. Date of interview	DD/MM/YYYY
3. Data collector ID	0-99
4. What is your age, in years?	0-99
5. Record the patient's sex.	1. Male<br>2. Female
6. Study ID	Numeric ID
7. Is the participant a contact of a newly diagnosed index case?	1. Yes<br>2. No
8. Has informed consent been provided by the participant if greater than or equal to 18 years of age?	1. Yes<br>2. No
9. Has parental consent and adolescent/child assent been provided if the participant is less than 18years of age?	1. Yes<br>2. No
10. What is the relationship of this contact to the index case?	1. Parent<br>2. Child<br>3. Sibling<br>4. Grandparent<br>5. Grandchild<br>6. Other family member<br>7. Unrelated household member<br>77. Don't know<br>88. Refuse
11. Which of the following symptoms do you currently have? *Select all that apply.*	1. Cough<br>2. Fever<br>3. Weight loss (more than 5 kg, or enough to make my clothes loose)<br>4. Drenching sweats at night<br>5. Pain in my chest<br>6. Pain in another part of my body<br>7. Skin problem (for example, rash)<br>8. Stomach or intestinal problem (for example, nausea or diarrhea)<br>9. Genital or urinary problem (for example, pain on urination, genital lesion)<br>10. None of the above<br>11. No symptoms
12. Which of your symptoms did you notice first? *Select only one.*	1. Cough<br>2. Fever

	3. Weight loss (more than 5 kg, or enough to make my clothes loose)<br>4. Drenching sweats at night<br>5. Pain in my chest<br>6. Pain in another part of my body<br>7. Skin problem (for example, rash)<br>8. Stomach or intestinal problem (for example, nausea or diarrhea)<br>9. Genital or urinary problem (for example, pain on urination, genital lesion)<br>10. None of the above
13. How long have you had that symptom?	0-10 years, 0-12 months, 0-4 weeks, 0-7 days<br>99 for unknown
14. If you had fever, cough, weight loss, or night sweats, but they were not the first symptom you noticed, how long did you have them?	0-10 years, 0-12 months, 0-4 weeks, 0-7 days<br>77 Unknown
15. How long ago was the last time that you visited a health clinic, for any reason?	0-10 years, 0-12 months, 0-4 weeks, 0-7 days<br>66 never<br>77 Unknown
16. In which village or town do you live?	(responses based on available towns)
17. What is the highest grade of education that you have attained?	0. None<br>1-12. Based on grades<br>13. Any postgraduate education<br>14. Attained postgraduate degree
18. Which of the following best describes your occupation?	1. Self-employed<br>2. Student<br>3. Salaried worker<br>4. Occasional work (piece jobs)<br>5. Unemployed but able to work<br>6. Unemployed and unable to work
19. Not including you, how many people in your household work?	0-20
20. If you had to sell all of your belongings and all of your household members' belongings (including your house, land, and any other valuables), how much money would you be able to get for your entire household's belongings?	R 0 – 1,000,000
21. From your perspective how much as your household member's having TB affected the household financially?	1. No impact<br>2. Little impact<br>3. Moderate impact<br>4. Serious impact<br>5. Very serious impact

22. Do you smoke tobacco?	1. Yes<br>2. Not currently, but formerly<br>3. No, never<br>88. Refused
23. For how many years have you smoked/did you smoke?	0-99 years<br>77 unknown
24. About how many cigarettes (or their equivalent) do you smoke, or did you smoke, each day?	0-999
25. Do you have any other medical conditions? Please check all that apply.	1. Obstructive lung disease (asthma or COPD)<br>2. Diabetes<br>3. Gastroesophageal reflux<br>4. Seasonal allergies<br>5. Silicosis or other destructive lung disease<br>6. Lung cancer<br>7. Any other form of cancer<br>8. High blood pressure<br>9. High cholesterol<br>10. HIV or AIDS<br>11. Other<br>88. Refused
26. Do you currently have TB?	1. Yes<br>2. No<br>77. Don't know
27. On what date were you diagnosed with TB?	DD/MM/YYYY
28. Are you taking treatment for TB?	1. Yes<br>2. No, completed<br>3. No, did not complete<br>77. Don't know
29. Have you ever been diagnosed with TB in the past?	1. Yes<br>2. No<br>9. Don't know
30. Approximately how long has it been since you were treated for TB in the past, if you ever were? Do not include the current episode.	0-99 years
31. Do you know your HIV status, and if you are comfortable to w ill you please share it with me?	1. Positive<br>2. Negative<br>77. Unknown<br>88. Refused
32. How long ago was the last time you were tested for HIV?	0-10 years, 0-12 months, 0-4 weeks, 0-7 days<br>66 never<br>77 n/a or unknown
33. Are you currently taking antiretrovirals for	1. Yes

your HIV?	2. No<br>77. Don't know or Not applicable
34. If you were to develop a mild cough, how long would it likely be before you saw a doctor or other healthcare professional for a diagnosis?	0-12 months, 0-4 weeks, 0-7 days<br>77 n/a or unknown
35. Once you did decide to see a doctor or other professional, where would you go?	1. Pharmacy<br>2. Traditional healer<br>3. This clinic<br>4. A different public clinic<br>5. Private clinic<br>6. Public hospital<br>7. Private hospital8. Other
36. How long have you lived in your current household?	1. Less than 1 year<br>2. 1-2 years<br>3. 3-5 years<br>4. More than 5 years<br>77. Unknown
37. Was a sputum sample obtained?	1. Yes<br>2. No
38. Date sputum sample taken	DD/MM/YYY
39. Are you comfortable with being visited by our staff and tested for TB?	1. Yes<br>2. No<br>88. Refused
40. Did the participant consent to HIV counselling and testing?	1. Yes<br>2. No<br>3. N/A: known positive
41. Result of HIV testing	1. Negative<br>2. Positive<br>3. Discordant (confirmatory test done)<br>4. Unable to complete

Form 3: Household Contact Arm Index Interview
To be completed for every consenting index patient in the household contact tracing arm

Question	Response Elements
1. Name of facility	1-56 (depending on specific facilities included)
2. Date of interview	DD/MMM/YYYY
3. Data collector ID	0-99
4. What is your age, in years?	0-99
5. Record the participant's sex.	1. Male<br>2. Female
6. Study ID	Numeric study ID
7. Is the participant a newly diagnosed TB case?	1. Yes<br>2. No
8. Has informed consent been provided by the participant if >18 years of age?	1. Yes<br>2. No
9. Has parental consent and adolescent/child assent been provided if participant is <18 years of age?	1. Yes<br>2. No
10. Have you started taking your TB treatment?	1. Yes<br>2. No, have not been back to clinic to initiate<br>3. No, received but has not started
11. Date of TB treatment initiation	DD/MM/YYYY
12. I want you to think back to the day when you originally came to clinic and were asked to give a specimen for a TB test (by coughing in a cup). What was the primary reason that you went to clinic that day?	1. I have a long-term condition such as HIV or cancer, and I was coming for regular care.<br>2. I had a cough, fever, sweats, or weight loss, and was coming for a diagnosis of those symptoms.<br>3. I was coming for diagnosis or treatment of other short-term symptoms.<br>4. I was coming for a regular check-up (or for antenatal care), not because I thought I was sick at all.<br>5. I was coming for medications, advice, or some other reason not listed above.<br>6. I was accompanying someone else.<br>77. No answer/don't know
13. On that day, which of the following symptoms did you have? *Select all that apply.*	1. Cough<br>2. Fever<br>3. Weight loss (more than 5 kg, or enough to make my clothes loose)<br>4. Drenching sweats at night<br>5. Pain in my chest<br>6. Pain in another part of my body<br>7. Skin problem (for example, rash)

	8. Stomach or intestinal problem (for example, nausea or diarrhea)<br>9. Genital or urinary problem (for example, pain on urination, genital lesion)<br>10. None of the above<br>11. No symptoms (for example, came for routine checkup of accompanying someone else)
14. Which of your symptoms did you notice first? *Select only one.*	1. Cough<br>2. Fever<br>3. Weight loss (more than 5 kg, or enough to make my clothes loose)<br>4. Drenching sweats at night<br>5. Pain in my chest<br>6. Pain in another part of my body<br>7. Skin problem (for example, rash)<br>8. Stomach or intestinal problem (for example, nausea or diarrhea)<br>9. Genital or urinary problem (for example, pain on urination, genital lesion)<br>10. None of the above
15. How long had you had that symptom before you came to clinic that day?	0-10 years, 0-12 months, 0-4 weeks, 0-7 days<br>77 for unknown
16. If you had fever, cough, weight loss, or night sweats, but they were not the first symptom you noticed, how long did you have them?	0-10 years, 0-12 months, 0-4 weeks, 0-7 days<br>77 Unknown
17. To what extent has your current TB illness affected your household **financially**?	1. No impact<br>2. Little impact<br>3. Moderate impact<br>4. Serious impact<br>5. Very serious impact
18. On the day you were asked to give a specimen for a TB test (by coughing in a cup), approximately how long did it take you to get to clinic?	0-24 hours, 0-60 minutes
19. What was your primary mode of transport (meaning the type of transport that took you the longest distance)?	1. Walking<br>2. Bus<br>3. Taxi<br>4. Motorbike<br>5. Car<br>6. Tuk-tuk<br>7. Bicycle<br>8. Other
20. How much do you estimate that a one-way trip to the clinic cost you?	R 0 – 10,000

21. On that day, how much time did it take for you to get to the clinic, starting from when you left your house?	0-24 hours, 0-60 minutes
22. On that day, how much TOTAL time did you spend for your visit to clinic, from the time that you left your home until the time you returned home?	0-24 hours, 0-60 minutes
23. Please estimate the amount of money you and your household members spent on that visit to the clinic that you would not have had to spend if you never came to clinic. For example: transportation, childcare, any food you had to purchase that you would not have bought at home or work, and the cost of any medicines. Please include the value of any goods or services you have had to sell or exchange, and any money that you have had to borrow from someone else, as well as the value of any missed work. Please also include the costs for anyone else who might have accompanied you to clinic today.	R 0 – 10,000
24. Before that visit, how long had it been since you had last visited this clinic (or another clinic like this one), for any reason?	0-10 years, 0-12 months, 0-4 weeks, 0-7 days<br>66 never<br>77 n/a or unknown
25. How many visits did you make to seek care or advice for TB symptoms (cough, fever, night sweats, or weight loss) including being admitted to the hospital? (Include informal providers such as herbalists, chemists, and formal providers such as emergency care, private clinics, government health centers)	0-6+ visits
26. Taking all of these visits together, plus any other costs to manage your TB symptoms (for example: cough medicine, special foods such as meats or energy drinks, vitamins, caregivers), please estimate the TOTAL amount of money you and your household members have had to spend to manage your symptoms since they started, that you would not have had to spend if you never got sick. Please include the value of any goods or services you have had to sell or exchange, and any money that you have had to borrow from someone else.	R 0 – 10,000
27. In addition to what you have spent above,	R 0 – 10,000

please also estimate the TOTAL amount of money that you would have earned, that you did not earn because of your TB symptoms. Please include both lost time from work because you were sick, and time that you missed from working because you had to visit clinics or see other healthcare providers. As above, please also include the value of any goods or services you have had to sell or exchange, and any money that you have had to borrow from someone else.	
28. In which village or town do you live?	(responses based on available towns)
29. What is the highest grade of education that you have attained?	0. None<br>1-12. Based on grades<br>13. Any postgraduate education<br>14. Attained postgraduate degree
30. Which of the following best describes your occupation?	1. Self-employed<br>2. Student<br>3. Salaried worker<br>4. Occasional work (piece jobs)<br>5. Unemployed but able to work<br>6. Unemployed and unable to work
31. Not including you, how many people in your household work?	0-20
32. Do you smoke tobacco?	1. Yes<br>2. Not currently, but formerly<br>3. No, never<br>88. Refused
33. For how many years have you smoked/did you smoke?	0-99 years<br>77 unknown
34. About how many cigarettes (or their equivalent) do you smoke, or did you smoke, each day?	0-999

35. Do you have any other medical conditions? Please check all that apply.	0. None<br>1. Obstructive lung disease (asthma or COPD)<br>2. Diabetes<br>3. Gastroesophageal reflux<br>4. Seasonal allergies<br>5. Silicosis or other destructive lung disease<br>6. Lung cancer<br>7. Any other form of cancer<br>8. High blood pressure<br>9. High cholesterol<br>10. HIV or AIDS<br>11. Other<br>88. Refused
36. Have you ever been diagnosed with TB in the past?	1. Yes<br>2. No<br>77. Don't know
37. Approximately how long has it been since you were treated for TB in the past? Do not include the current episode.	0-99 years
38. Do you know your HIV status, and if you are comfortable to will you please share it with me?	1. Positive<br>2. Negative<br>77. Unknown<br>88. Refused
39. How long ago was the last time you were tested for HIV?	0-10 years, 0-12 months, 0-4 weeks, 0-7 days<br>66 never<br>77 n/a or unknown
40. Are you currently taking antiretrovirals for your HIV?	1. Yes<br>2. No<br>77. Don't know
41. If you were to develop a mild cough, how long would it likely be before you saw a doctor or other healthcare professional for a diagnosis?	0-12 months, 0-4 weeks, 0-7 days<br>77 n/a or unknown
42. Once you did decide to see a doctor or other professional, where would you go?	1. Pharmacy<br>2. Traditional healer<br>3. This clinic<br>4. A different public clinic<br>5. Private clinic<br>6. Public Hospital<br>7. Private hospital<br>8. Other
43. What is your feeling about getting tested for TB, even if you were coming to clinic for a completely different reason?	1. I am glad to be tested.<br>2. I am willing to be tested.<br>3. I don't care whether I am tested or not.

	4. I prefer not to be tested.<br>5. I would refuse to be tested.
44. How many people, not including yourself, live in your household? Please include everyone- adults and children – who sleep here at least one night a week. Please do not include guests and temporary visitors.	0-99
45. Does anyone in your house have TB, or been told they have TB in the last 2 years?	1. Yes<br>2. No77. Don't know
46. Who is the head of your household?	1. Myself<br>2. Male >60 years<br>3. Male 18-60 years<br>4. Male child <18 years<br>5. Female >60 years<br>6. Female 18-60 years<br>7. Female child <18 years
47. If you are not the head of the household, what is the level of education of the head of the household?	0. None<br>1-12. Based on grades<br>13. Any postgraduate education<br>14. Attained postgraduate degree
48. How long have you lived in your current household?	1. Less than 6 months<br>1. 6 months to 1 year<br>2. 1 year to 5 years<br>3. More than 5 years<br>77 Unknown
49. What is your household's estimated monthly income from each of the following sources:<br>a. Regular employment<br>b. Casual work<br>c. Grant/pensions<br>d. Other sources (traded goods, investments, etc.)	 <br>R 0 – 100,000<br>R 0 – 100,000<br>R 0 – 100,000<br>R 0 – 100,000<br><br>77. Unknown<br>88. Refused
50. Please think back to the time before you were sick. Can you estimate your monthly income at that time? If you were paid in-kind or received payment not in cash (for example, for crops), please estimate how much cash you would receive if you sold those goods on the market today.	R 0 – 100,000
51. If you had to sell all of your belongings and all	R 0 – 1,000,000

of your household members' belongings (including your house, land, and any other valuables), how much money would you be able to get for your entire household's belongings?	
52. Are you comfortable with your household members being visited by our staff and tested for TB?	1. Yes<br>2. No<br>77. Don't know
53. Did the participant consent to HIV counselling and testing?	1. Yes<br>2. No<br>3. N/A: known positive
54. Result of HIV testing	1. Negative<br>2. Positive<br>3. Discordant (confirmatory test done)<br>4. Unable to complete

Form 6: Incentive-Based Contact Arm Contact Interview

To be completed for every consenting contact in the incentive-based contact tracing arm

**Question**	**Response Elements**
1. Name of facility	1-56 (depending on specific facilities included)
2. Date of interview	DD/MM/YYYY
3. Data collector ID	0-99
4. What is your age, in years?	0-99
5. Record the patient's sex.	1. Male<br>2. Female
6. Study ID	Numeric ID
7. Is the participant a contact of a newly diagnosed index case?	1. Yes<br>2. No
8. Has informed consent been provided by the participant if >18 years of age?	1. Yes<br>2. No
9. Has parental consent and adolescent/child assent been provided if the participant is <18 years of age?	1. Yes<br>2. No
10. How do you know the person who gave you the coupon to come to the clinic for TB screening?	1. Family member from household<br>2. Non-family member from household<br>3. Family member from outside household<br>4. Friend or acquaintance<br>5. Work colleague<br>77. Don't know<br>88. Refuse
11. How long have you known this person for?	1. 5 years or more<br>2. 1-4 years<br>3. 6-11 months<br>4. Less than 6 months<br>5. Don't know<br>88. Refuse
12. Which of the following symptoms do you currently have? *Select all that apply.*	1. Cough<br>2. Fever<br>3. Weight loss (more than 5 kg, or enough to make my clothes loose)<br>4. Drenching sweats at night<br>5. Pain in my chest<br>6. Pain in another part of my body<br>7. Skin problem (for example, rash)<br>8. Stomach or intestinal problem (for example, nausea or diarrhea)<br>9. Genital or urinary problem (for example, pain on urination, genital lesion)

	10. None of the above<br>11. No symptoms
13. Which of your symptoms did you notice first?<br>*Select only one.*	1. Cough<br>2. Fever<br>3. Weight loss (more than 5 kg, or enough to make my clothes loose)<br>4. Drenching sweats at night<br>5. Pain in my chest<br>6. Pain in another part of my body<br>7. Skin problem (for example, rash)<br>8. Stomach or intestinal problem (for example, nausea or diarrhea)<br>9. Genital or urinary problem (for example, pain on urination, genital lesion)<br>10. Don't know
14. How long have you had that symptom?	0-10 years, 0-12 months, 0-4 weeks, 0-7 days<br>77 for unknown
15. If you had fever, cough, weight loss, or night sweats, but they were not the first symptom you noticed, how long did you have them?	0-10 years, 0-12 months, 0-4 weeks, 0-7 days<br>77 for n/a or unknown
16. How long ago was the last time that you visited a health clinic, for any reason?	0-10 years, 0-12 months, 0-4 weeks, 0-7 days<br>66 never<br>77 n/a or unknown
17. Approximately how long did it take you to get to clinic today?	0-24 hours, 0-60 minutes
18. How much TOTAL time do you expect to spend for your visit to clinic today, from the time that you left your home until the time you expect to return home?	0-24 hours, 0-60 minutes
19. What was your primary mode of transport (meaning the type of transport that took you the longest distance)?	1. Walking<br>2. Bus<br>3. Taxi<br>4. Motorbike<br>5. Car<br>6. Tuk-tuk<br>7. Bicycle<br>9. Other
20. How much do you estimate that a one-way trip to the clinic cost you?	R 0 – 10,000
21. Please estimate the amount of money you and your household members have had to spend on today's visit to clinic, that you would not have had	R 0 – 10,000

to spend if you never came to clinic. For example: transportation, childcare, any food you had to purchase that you would not have bought at home or work, and the cost of any medicines. Please include the value of any goods or services you have had to sell or exchange, and any money that you have had to borrow from someone else, as well as the value of any missed work. Please also include the costs for anyone else who might have accompanied you to clinic today.	
22. In which village or town do you live?	(responses based on available towns)
23. What is the highest grade of education that you have attained?	0. None<br>1-12. Based on grades<br>13. Any postgraduate education<br>14. Attained postgraduate degree
24. Which of the following best describes your occupation?	1. Self-employed<br>2. Student<br>3. Salaried worker<br>4. Occasional work (piece jobs)<br>5. Unemployed but able to work<br>6. Unemployed and unable to work
25. Not including you, how many people in your household work?	0-20
26. If you had to sell all of your belongings and all of your household members' belongings (including your house, land, and any other valuables), how much money would you be able to get for your entire household's belongings?	R 0 – 1,000,000
27. If the person who gave you your coupon is your household member, how has their having TB affected them or their household financially?	1. No impact<br>2. Little impact<br>3. Moderate impact<br>4. Serious impact<br>5. Very serious impact
28. Do you smoke tobacco?	1. Yes<br>2. Not currently, but formerly<br>3. No, never<br>88. Refused
29. For how many years have you smoked/did you smoke?	0-99 years<br>77 unknown
30. About how many cigarettes (or their equivalent) do you smoke, or did you smoke, each day?	0-999

31. Do you have any other medical conditions? Please check all that apply.	1. Obstructive lung disease (asthma or COPD)<br>2. Diabetes<br>3. Gastroesophageal reflux<br>4. Seasonal allergies<br>5. Silicosis or other destructive lung disease<br>6. Lung cancer<br>7. Any other form of cancer<br>8. High blood pressure<br>9. High cholesterol<br>10. HIV or AIDS<br>11. Other<br>88. Refused
32. Do you currently have TB?	1. Yes<br>2. No<br>77. Don't know
33. On what date were you diagnosed with TB?	DD/MM/YYYY
34. Are you taking treatment for TB?	1. Yes<br>2. No, completed<br>3. No, did not complete<br>77. Don't know
35. Have you ever been diagnosed with TB in the past?	1. Yes<br>2. No<br>77. Don't know
36. Approximately how long has it been since you were treated for TB in the past, if you ever were? Do not include the current episode.	0-99 years
37. Do you know your HIV status, and if you are comfortable to will you please share it with me?	1. Positive<br>2. Negative<br>3. Unknown<br>88. Refused
38. How long ago was the last time you were tested for HIV?	0-10 years, 0-12 months, 0-4 weeks, 0-7 days<br>66 never<br>77 n/a or unknown
39. Are you currently taking antiretrovirals for your HIV?	1. Yes<br>2. No<br>77. Don't know or Not applicable
40. If you were to develop a mild cough, how long would it likely be before you saw a doctor or other healthcare professional for a diagnosis?	0-12 months, 0-4 weeks, 0-7 days<br>77 n/a or unknown
41. Once you did decide to see a doctor or other professional, where would you go?	1. Pharmacy<br>2. Traditional healer<br>3. A clinic that is staffed by medical doctors<br>4. Hospital

	5. Village or private clinic<br>6. Other
42. How long have you lived in your current household?	0-98 years, 0-12 months, 0-4 weeks, 0-7 days<br>77 n/a or unknown
43. How many people, including yourself, live in your household? Please include everyone- adults and children – who sleep here at least one night a week. Please do not include guests and temporary visitors.	0-99
44. Does anyone in your house have TB, or been told they have TB in the last 2 years?	1. Yes<br>2. No<br>77. Don't know
45. Who is the head of your household?	1. Myself<br>2. Male >60 years<br>3. Male 18-60 years<br>4. Male child <18 years<br>5. Female >60 years<br>6. Female 18-60 years<br>7. Female child <18 years
46. If you are not the head of the household, what is the level of education of the head of the household?	0. None<br>1-12. Based on grades<br>13. Any postgraduate education<br>14. Attained postgraduate degree
47. What is your household's estimated monthly income from each of the following sources:<br>a. Regular employment<br>b. Casual work<br>c. Grant/pensions<br>d. Other sources (traded goods, investments, etc.)	 <br>R 0 – 100,000<br>R 0 – 100,000<br>R 0 – 100,000<br>R 0 – 100,000<br><br>77. Unknown<br>88. Refused
48. What is your feeling about getting this voucher, to get tested for TB?	1. I am glad to get this voucher and get tested.<br>2. I am willing to get this voucher and get tested.<br>3. I am unhappy about getting this voucher and getting tested.<br>4. I refuse to get tested.
49. Was a sputum sample obtained?	1. Yes<br>2. No
50. Date sputum sample taken	DD/MM/YYYY
51. Did the participant consent to HIV counselling and testing?	1. Yes<br>2. No<br>3. N/A: known positive

52. Result of HIV testing	1. Negative<br>2. Positive<br>3. Discordant (confirmatory test done)<br>4. Unable to complete

Form 5: Incentive-Based Contact Arm Index Interview

To be completed for every consenting index patient in the incentive-based contact tracing arm

**Question**	**Response Elements**
1. Name of facility	1-56 (depending on specific facilities included)
2. Date of interview	DD/MM/YYYY
3. Data collector ID	0-99
4. What is your age, in years?	0-99
5. Record the patient's sex.	1. Male<br>2. Female
6. Study ID	Numeric ID
7. Is the participant a newly diagnosed TB case?	1. Yes<br>2. No
8. Has informed consent been provided by the participant if >18 years of age?	1. Yes<br>2. No
9. Has parental consent and adolescent/child assent been provided if the participant is <18 years of age?	1. Yes<br>2. No
10 Have you started taking your TB treatment?	1. Yes<br>2. No, have not been back to clinic to initiate<br>3. No, received but have not started
11. Date of TB treatment initiation	DD/MM/YYYY
12. I want you to think back to the day when you originally came to clinic and were asked to give a specimen for a TB test (by coughing in a cup). What was the primary reason that you went to clinic that day?	1. I have a long-term condition such as HIV or cancer, and I was coming for regular care.<br>2. I had a cough, fever, sweats, or weight loss, and was coming for a diagnosis of those symptoms.<br>3. I was coming for diagnosis or treatment of other short-term symptoms.<br>4. I was coming for a regular check-up (or for antenatal care), not because I thought I was sick at all.<br>5. I was coming for medications, advice, or some other reason not listed above.<br>6. I was accompanying someone else.<br>77. No answer/don't know
13. On that day, which of the following symptoms did you have? *Select all that apply.*	1. Cough<br>2. Fever<br>3. Weight loss (more than 5 kg, or enough to make my clothes loose)<br>4. Drenching sweats at night<br>5. Pain in my chest<br>6. Pain in another part of my body<br>7. Skin problem (for example, rash)<br>8. Stomach or intestinal problem (for example,

	nausea or diarrhea)<br>9. Genital or urinary problem (for example, pain on urination, genital lesion)<br>10. None of the above<br>11. No symptoms (for example, came for routine checkup or accompanying someone else)
14. Which of your symptoms did you notice first?<br>*Select only one.*	1. Cough<br>2. Fever<br>3. Weight loss (more than 5 kg, or enough to make my clothes loose)<br>4. Drenching sweats at night<br>5. Pain in my chest<br>6. Pain in another part of my body<br>7. Skin problem (for example, rash)<br>8. Stomach or intestinal problem (for example, nausea or diarrhea)<br>9. Genital or urinary problem (for example, pain on urination, genital lesion)<br>77. Don't know
15. How long had you had that symptom before you came to clinic that day?	0-10 years, 0-12 months, 0-4 weeks, 0-7 days<br>77 for unknown
16. If you had fever, cough, weight loss, or night sweats, but they were not the first symptom you noticed, how long did you have them?	0-10 years, 0-12 months, 0-4 weeks, 0-7 days<br>77 for n/a or unknown
17. To what extent has your current TB illness affected your household **financially**?	1. No impact<br>2. Little impact<br>3. Moderate impact<br>4. Serious impact<br>5. Very serious impact
18. On that day you were asked to give a specimen for a TB test (by coughing in a cup), approximately how long did it take you to get to clinic?	0-24 hours, 0-60 minutes
19. What was your primary mode of transport (meaning the type of transport that took you the longest distance)?	1. Walking<br>2. Bus<br>3. Taxi<br>4. Motorbike<br>5. Car<br>6. Tuk-tuk<br>7. Bicycle<br>9. Other
20. How much do you estimate that a one-way trip to the clinic cost you?	R 0 – 10,000
21. On that day, how much time did it take for you	0-24 hours, 0-60 minutes

to get to the clinic, starting from when you left your house?	
22. On that day, how much TOTAL time did you spend for your visit to clinic, from the time that you left your home until the time you returned home?	0-24 hours, 0-60 minutes
23. Please estimate the amount of money you and your household members spent on that visit to the clinic that you would not have had to spend if you never came to clinic. For example: transportation, childcare, any food you had to purchase that you would not have bought at home or work, and the cost of any medicines. Please include the value of any goods or services you have had to sell or exchange, and any money that you have had to borrow from someone else, as well as the value of any missed work. Please also include the costs for anyone else who might have accompanied you to clinic today.	R 0 – 10,000
24. Before that visit, how long had it been since you had last visited this clinic (or another clinic like this one), for any reason?	0-10 years, 0-12 months, 0-4 weeks, 0-7 days<br>66 never<br>77 n/a or unknown
25. How many visits did you make to seek care or advice for TB symptoms (cough, fever, night sweats, or weight loss) including being admitted to the hospital? (Include informal providers such as herbalists, chemists, and formal providers such as emergency care, private clinics, government health centers)	0-6+ visits
26. Taking all of these visits together, plus any other costs to manage your TB symptoms (for example: cough medicine, special foods such as meats or energy drinks, vitamins, caregivers), please estimate the TOTAL amount of money you and your household members have had to spend to manage your symptoms since they started, that you would not have had to spend if you never got sick. Please include the value of any goods or services you have had to sell or exchange, and any money that you have had to borrow from someone else.	R 0 – 10,000
27. In addition to what you have spent above, please also estimate the TOTAL amount of money	R 0 – 10,000

that you would have earned, that you did not earn because of your TB symptoms. Please include both lost time from work because you were sick, and time that you missed from working because you had to visit clinics or see other healthcare providers. As above, please also include the value of any goods or services you have had to sell or exchange, and any money that you have had to borrow from someone else.	
28. In which village or town do you live?	(responses based on available towns)
29. What is the highest grade of education that you have attained?	0. None<br>1-12. Based on grades<br>13. Any postgraduate education<br>14. Attained postgraduate degree
30. Which of the following best describes your occupation?	1. Self-employed<br>2. Student<br>3. Salaried worker<br>4. Occasional work (piece jobs)<br>5. Unemployed but able to work<br>6. Unemployed and unable to work
31. Not including you, how many people in your household work?	0-20
32. Do you smoke tobacco?	1. Yes<br>2. Not currently, but formerly<br>3. No, never<br>88. Refused
33. For how many years have you smoked/did you smoke?	0-99 years<br>77 unknown
34. About how many cigarettes (or their equivalent) do you smoke, or did you smoke, each day?	0-999
35. Do you have any other medical conditions? Please check all that apply.	1. Obstructive lung disease (asthma or COPD)<br>2. Diabetes<br>3. Gastroesophageal reflux<br>4. Seasonal allergies<br>5. Silicosis or other destructive lung disease<br>6. Lung cancer<br>7. Any other form of cancer<br>8. High blood pressure<br>9. High cholesterol<br>10. HIV or AIDS<br>11. Other

	88. Refused
36. Have you ever been diagnosed with TB in the past?	1. Yes<br>2. No<br>77. Don't know
37. Approximately how long has it been since you were treated for TB in the past, if you ever were? Do not include the current episode.	0-99 years
38. Do you know your HIV status, and if you are comfortable to will you please share it with me?	1. Positive<br>2. Negative<br>3. Unknown<br>88. Refused
39. How long ago was the last time you were tested for HIV?	0-10 years, 0-12 months, 0-4 weeks, 0-7 days<br>66 never<br>77 n/a or unknown
40. Are you currently taking antiretrovirals for your HIV?	1. Yes<br>2. No<br>77. Don't know or Not applicable
41. If you were to develop a mild cough, how long would it likely be before you saw a doctor or other healthcare professional for a diagnosis?	0-12 months, 0-4 weeks, 0-7 days<br>77 n/a or unknown
42. Once you did decide to see a doctor or other professional, where would you go?	1. Pharmacy<br>2. Traditional healer<br>3. This clinic<br>4. A different public clinic<br>5. Private clinic<br>6. Public hospital<br>7. Private hospital<br>8. Other
43. What is your feeling about getting tested for TB, even if you were coming to clinic for a completely different reason?	1. I am glad to be tested.<br>2. I am willing to be tested.<br>3. I don't care whether I am tested or not.<br>4. I prefer not to be tested.<br>5. I would refuse to be tested.
44. How many people, not including yourself, live in your household? Please include everyone- adults and children – who sleep here at least one night a week. Please do not include guests and temporary visitors.	0-99
45. Does anyone in your house have TB, or been told they have TB in the last 2 years?	1. Yes<br>2. No<br>77. Don't know
46. Who is the head of your household?	1. Myself<br>2. Male >60 years

	3. Male 18-60 years<br>4. Male child <18 years<br>5. Female >60 years<br>6. Female 18-60 years<br>7. Female child <18 years
47. If you are not the head of the household, what is the level of education of the head of the household?	0. None<br>1-12. Based on grades<br>13. Any postgraduate education<br>14. Attained postgraduate degree
48. How long have you lived in your current household?	0-98 years, 0-12 months, 0-4 weeks, 0-7 days<br>77 n/a or unknown
49. What is your household's estimated monthly income from each of the following sources:<br>a. Regular employment<br>b. Casual work<br>c. Grant/pensions<br>d. Other sources (traded goods, investments, etc.)	 <br>R 0 – 100,000<br>R 0 – 100,000<br>R 0 – 100,000<br>R 0 – 100,000<br><br>77. Unknown<br>88. Refused
50. Please think back to the time before you were sick. Can you estimate your monthly income at that time? If you were paid in-kind or received payment not in cash (for example, for crops), please estimate how much cash you would receive if you sold those goods on the market today.	R 0 – 100,000
51. If you had to sell all of your belongings and all of your household members' belongings (including your house, land, and any other valuables), how much money would you be able to get for your entire household's belongings?	R 0 – 1,000,000
52. Are you comfortable giving vouchers to people you know, to get tested for TB?	1. Yes<br>2. No<br>77. Don't know
53. Did the participant consent to HIV counselling and testing?	1. Yes<br>2. No<br>3. N/A: known positive
54. Result of HIV testing	1. Negative<br>2. Positive<br>3. Discordant (confirmatory test done)

	4. Unable to complete
